# SARS-CoV-2 mRNA Vaccination Induces Reduced T-Cell Apoptosis in Patients with Solid Tumors

**DOI:** 10.3390/ijms27146173

**Published:** 2026-07-10

**Authors:** Ana Belda-Marco, Lucía Serrano-García, Andrés Moret, Carlos Fresneda-Portillo, María Victoria Domínguez-Márquez, Ana Comes-Raga, Beatriz Jávega, José-Enrique O’Connor, Juan Carlos Andreu-Ballester, Antonio Llombart-Cussac, María Leonor Fernández-Murga

**Affiliations:** 1 Clinical and Molecular Oncology Laboratory, Arnau de Vilanova Hospital, FISABIO, 46015 Valencia, Spain; 2 Department of Mathematics and Computation, Superior Polytechnic School, University of Burgos, 09006 Burgos, Spain; 3 Department of Quantitative Methods, Universidad Loyola Andalucía, 41704 Sevilla, Spain; 4 Department of Microbiology, Arnau de Vilanova Hospital, 46015 Valencia, Spain; 5 Department of Clinical Analysis, Arnau de Vilanova Hospital, 46015 Valencia, Spain; 6 Flow Cytometry Unit, Central Unit for Research in Medicine (UCIM), University of Valencia, 46010 Valencia, Spain; 7 Laboratory of Cytomics, Joint Research Unit CIPF-UVEG, Department of Biochemistry and Molecular Biology, University of Valencia, 46010 Valencia, Spain; 8 Parasitic Immunobiology and Immunomodulation Research Group (INMUNOPAR), Complutense University of Madrid, 28040 Madrid, Spain; 9 Oncology Department, Arnau de Vilanova Hospital, 46015 Valencia, Spain; 10 Translational Oncology Group, Health Sciences Faculty, University Studies Center (CEU) Cardenal Herrera University, 46115 Alfara del Patriarca, Spain

**Keywords:** mRNA vaccine, SARS-CoV-2, solid tumors, humoral response, neutralizing antibodies, T-cell differentiation, early apoptosis, αβ T cells, γδ T cells, NKT-like cells

## Abstract

Messenger RNA (mRNA) vaccines represent a transformative platform in vaccinology, with applications extending beyond SARS-CoV-2 to other infectious diseases and cancer immunotherapy. However, patients with solid tumors receiving active anticancer treatment were largely underrepresented in pivotal vaccination trials, limiting understanding of vaccine-induced immunity in this population. In this prospective exploratory study, we assessed humoral and cellular immune responses after two doses of SARS-CoV-2 mRNA vaccines in 39 patients with solid tumors undergoing active treatment. Blood samples were collected before vaccination and approximately two months after the second vaccine dose, prior to the next treatment cycle. Anti-spike IgG, neutralizing antibodies, receptor-binding domain (RBD) levels, interleukin-6 (IL-6), hematological parameters, immune cell subsets, T-cell differentiation, and early apoptosis in αβ and γδ T-cell subsets were analyzed. Vaccination induced a robust humoral response, with high post-vaccination anti-spike IgG levels (median 988.69 BAU/mL), 97.44% seropositivity, 96.88% true seroconversion among baseline IgG−/NAb− patients, and strong neutralizing antibody activity (median 85.73%). Hematological parameters and IL-6 levels remained broadly stable, suggesting no detectable increase in systemic inflammation during the study period. Cellular analyses identified a reduction in peripheral CD19+ B-cell frequencies and decreased early apoptosis, particularly in CD8+ T cells and CD3+CD56+ NKT-like cells. Although changes in T-cell frequencies and differentiation profiles were also observed, these findings were attenuated after exclusion of participants with possible prior SARS-CoV-2 exposure and should be interpreted as exploratory. Overall, these results show that patients with solid tumors receiving active treatment can mount robust humoral responses to SARS-CoV-2 mRNA vaccination and suggest measurable post-vaccination changes in lymphocyte dynamics, including reduced early T-cell apoptosis.

## 1. Introduction

Messenger RNA (mRNA) vaccines represent a transformative advance in vaccinology. This platform utilizes lipid nanoparticle (LNP)-encapsulated nucleoside-modified mRNA to direct host cells to produce specific antigens, thereby eliciting both humoral and cellular immune responses [1]. The LNP carrier itself possesses intrinsic adjuvant activity, promoting T follicular helper cell differentiation, germinal center B-cell responses, and long-lived plasma cell formation [2,3]. The clinical success of BNT162b2 (Pfizer-BioNTech) and mRNA-1273 (Moderna) against Severe Acute Respiratory Syndrome Coronavirus 2 (SARS-CoV-2) demonstrated the remarkable efficacy of this platform, achieving high protection rates against symptomatic COVID-19 in the general population and becoming a cornerstone of pandemic control [4,5].

Beyond COVID-19, the mRNA-LNP platform has rapidly expanded into a broader vaccine development pipeline. Currently, mRNA vaccines are in advanced clinical development against influenza (phase 3), respiratory syncytial virus (RSV)—with mRNA-1345 (mRESVIA) receiving FDA approval in May 2024—cytomegalovirus, Epstein–Barr virus, Zika, and HIV, among other pathogens [6,7,8]. In parallel, the platform is being actively explored for therapeutic cancer vaccines, leveraging its ability to encode tumor-specific neoantigens and stimulate antitumor immunity [9,10]. This expanding landscape underscores the importance of understanding how mRNA vaccines interact with the immune system across diverse clinical populations, including immunocompromised hosts.

Patients with cancer represent a particularly relevant population for studying mRNA vaccine immunogenicity. The emergence of SARS-CoV-2 in late 2019 triggered a global health crisis with profound consequences for patients with cancer, who face increased risk of severe disease and adverse outcomes due to tumor-related and treatment-related immune dysfunction [11,12,13]. Among oncology patients, individuals with solid tumors undergoing active anticancer treatment—including cytotoxic chemotherapy, immune checkpoint inhibitors, and targeted agents—may have compromised immune competence [14,15]. Because of their elevated clinical risk, patients with cancer were prioritized in vaccination campaigns, despite their underrepresentation in pivotal vaccine trials, which has limited the available evidence on vaccine immunogenicity and protection in this setting [16].

Evidence accumulated in recent years suggests that vaccine responses in patients with solid tumors may differ quantitatively and qualitatively from those observed in healthy individuals. Meta-analyses have reported that seroconversion rates after COVID-19 vaccination are lower in patients with solid malignancies than in healthy controls, especially after the first dose, although this gap narrows substantially after subsequent doses, with seroconversion rates reaching approximately 90–95% after two vaccine doses [16,17,18]. The VOICE trial, a prospective multicenter non-inferiority study, demonstrated that the SARS-CoV-2-binding antibody response in patients receiving immunotherapy, chemotherapy, or chemoimmunotherapy for solid tumors was non-inferior to that of individuals without cancer, and proposed a potential threshold of 300 binding antibody units (BAU)/mL to discriminate adequate from suboptimal responders [19]. More recently, long-term data have shown that patients with solid malignancies receiving chemotherapy can effectively generate durable humoral and cellular responses at 6 and 12 months post-vaccination [20].

Protection against SARS-CoV-2 depends not only on antibody production but also on the induction and maintenance of cellular immune responses. However, studies evaluating T-cell responses in vaccinated cancer patients have shown that, although many patients mount detectable cellular immunity, the magnitude and coordination of these responses may differ from those of immunocompetent individuals, with lower CD4+ and CD8+ T-cell response rates reported in patients receiving active treatment [21,22]. Memory CD8+ T-cell and B-cell responses have been linked to protection after mRNA vaccination, supporting the relevance of cellular immune remodeling as a determinant of vaccine responsiveness [23]. In particular, the balance between naïve, memory, and terminally differentiated T-cell subsets may reflect the quality and durability of vaccine-induced cellular immunity, yet these differentiation dynamics remain largely uncharacterized in patients with solid tumors receiving active treatment.

Likewise, little is known about how mRNA vaccination may influence lymphocyte survival in these patients. The transition from effector to memory T-cell states is critically regulated by the balance between pro- and anti-apoptotic signals; long-lived memory T cells are characteristically refractory to apoptosis through upregulation of Bcl-2 family members and downregulation of pro-apoptotic molecules [24,25,26]. Apoptosis patterns may therefore provide additional mechanistic insight into immune adaptation after vaccination, yet this dimension has been scarcely explored in the context of mRNA vaccination in oncology patients.

Despite these advances, important gaps remain in our understanding of vaccine-induced immunity in patients with solid tumors under active anticancer treatment. In particular, the behavior of distinct T-cell compartments, including conventional αβ T cells and unconventional γδ T cells, remains insufficiently characterized in this setting. γδ T cells bridge innate and adaptive immunity and play recognized roles in both antiviral defense and tumor surveillance [27,28], yet their response to mRNA vaccination in oncology patients has received limited attention. Our group previously reported that patients with solid tumors receiving systemic anticancer therapy at our institution had low SARS-CoV-2 seroprevalence during the first pandemic wave but experienced a high rate of potentially COVID-19-related symptoms, highlighting the clinical relevance of understanding vaccine-induced immunity in this population [29]. In a subsequent study in a non-oncological cohort of healthy volunteers using the same flow cytometric methodology, we showed that baseline γδ T-cell levels and pre-vaccination apoptosis were associated with post-vaccination SARS-CoV-2 infection risk, providing a complementary reference framework from immunocompetent individuals [30]. To our knowledge, simultaneous assessment of humoral responses, T-cell differentiation dynamics, and apoptosis patterns in αβ and γδ T-cell populations has been scarcely explored in patients with solid tumors receiving active anticancer treatment after mRNA SARS-CoV-2 vaccination.

In the present prospective exploratory study, we evaluated humoral and cellular immune responses after the second dose of mRNA-based SARS-CoV-2 vaccines in patients with solid tumors undergoing active anticancer therapy. Specifically, we assessed anti-SARS-CoV-2 antibody responses, circulating immune cell subsets, T-cell differentiation stages, and early apoptosis in αβ and γδ T-cell populations, and explored the influence of clinical and treatment-related variables on post-vaccination immune profiles. Understanding these broader immune effects of mRNA vaccination in cancer patients is relevant as the mRNA platform expands beyond COVID-19 into new infectious disease vaccines and therapeutic cancer applications, where the interaction between vaccine-induced immunity and the tumor-altered immune context may influence clinical outcomes.

## 2. Results

### 2.1. Patient Cohort and Clinical Characteristics

Recruitment was initially constrained by COVID-19-related public health restrictions, disease progression, vaccine refusal, and refusal to provide post-vaccination samples among oncology patients. The final cohort included 39 patients, comprising 18 males (46.15%) and 21 females (53.85%), with a median age of 58 years (range: 29–76). Breast and ovarian tumors were the most frequent malignancies (46.15%), followed by gastrointestinal (23.08%), respiratory (17.95%) and genitourinary cancers (12.82%). Most patients had stage III disease (33.33%) and were receiving chemotherapy (71.79%) at the time of inclusion.

Overall, 22 patients (56.41%) had at least one comorbidity, with hypertension (30.77%) and dyslipidemia (15.39%) being the most frequent. Baseline SARS-CoV-2 humoral immunity status was also assessed. Thirty-two patients (82.05%) were negative for both anti-SARS-CoV-2 IgG and neutralizing antibodies, whereas five patients (12.82%) had detectable baseline IgG and neutralizing antibodies, and two additional patients (5.13%) showed neutralizing activity despite negative IgG. These patients had no documented history of COVID-19 and tested negative by PCR at recruitment, suggesting possible previous unrecognized asymptomatic SARS-CoV-2 exposure. A detailed summary of the baseline clinical and demographic characteristics of the study population is presented in Table 1.

### 2.2. Vaccination Outcomes and Humoral Response

All 39 patients received two doses of mRNA-based SARS-CoV-2 vaccines. Post-vaccination symptoms were generally mild and lasted 24–48 h in most cases. Clinical vaccination outcomes are summarized in Table S1.

**Figure 1:**
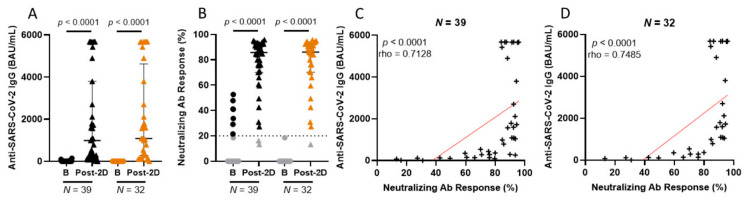
Humoral response to SARS-CoV-2 mRNA vaccination. Anti-spike IgG levels and neutralizing antibody activity were assessed at Baseline (B) and approximately two months after the second vaccine dose (Post-2D) in the complete cohort (*N* = 39) and in the restricted cohort (*N* = 32). Changes in (**A**) anti-spike IgG levels and (**B**) neutralizing antibody activity. The dotted line indicates the positivity threshold of this assay (20%) Values below this threshold are shown in gray. (**C**,**D**) Correlation between post-vaccination anti-spike IgG levels and neutralizing antibody activity in the complete and restricted cohorts, respectively. Paired comparisons were performed using the Wilcoxon signed-rank test, and correlations using Spearman’s rho. Displayed *p*-values are unadjusted. Ab: antibody; BAU: Binding antibody units.

Humoral response was assessed before vaccination (Baseline, B) and approximately two months after the second vaccine dose (Post-2D). Anti-spike IgG titers increased markedly after vaccination, rising from a median of 0 BAU/mL at baseline to 988.69 BAU/mL post-vaccination (*p* < 0.0001; r = 0.86) (Figure 1A, Table 2). Similarly, neutralizing antibody activity increased from 0% to 85.73% (*p* < 0.0001; r = 0.87) (Figure 1B, Table 2). Post-vaccination anti-spike IgG levels correlated strongly with neutralizing antibody activity (*p* < 0.0001) (Figure 1C). No significant associations were detected between the magnitude of the humoral response and sex, age, cancer type, anticancer therapy, disease stage, or number of comorbidities.

As described in Table 1, seven patients showed detectable baseline anti-SARS-CoV-2 IgG and/or neutralizing antibody activity, suggesting possible previous unrecognized SARS-CoV-2 exposure. To assess the potential influence of baseline humoral immunity, sensitivity analyses were performed after excluding these patients. In the restricted cohort (*N* = 32), the main findings remained unchanged: anti-spike IgG levels increased from 0 BAU/mL at baseline to 1082.71 BAU/mL post-vaccination (*p* < 0.0001; r = 0.86), and neutralizing antibody activity increased from 0% to 86.06% (*p* < 0.0001; r = 0.86) (Figure 1A,B). The correlation between anti-spike IgG levels and neutralizing activity was also preserved (*p* < 0.0001) (Figure 1D). These results indicate that the robust post-vaccination humoral response was not driven by patients with possible prior SARS-CoV-2 exposure.

Seropositivity was defined as the presence of detectable anti-spike IgG after vaccination, regardless of baseline antibody status. In contrast, seroconversion was defined as the transition from anti-spike IgG negativity at baseline to anti-spike IgG positivity after vaccination among patients without baseline humoral immunity. Accordingly, after vaccination, 38 of 39 patients (97.44%) were seropositive for anti-spike IgG. Among patients who were IgG−/NAb− at baseline (*N* = 32), 31 of 32 (96.88%) showed true seroconversion. Neutralizing antibody activity was also detected in 30 (93.75%) patients after vaccination; however, two patients with breast cancer receiving chemotherapy did not reach the positivity threshold for neutralizing antibodies (20%). In addition, serum RBD was detectable in four patients (10.26%) after vaccination, although this finding should be interpreted cautiously, as the present study was not designed to assess antigen persistence or clearance.

### 2.3. Hematological and Inflammatory Profile Before and After Vaccination

Routine hematological parameters and serum interleukin 6 (IL-6) levels were assessed to characterize the systemic clinical and inflammatory status of patients during the vaccination period. IL-6 was included as a circulating marker of systemic inflammation and immune activation, which may reflect both the underlying malignancy and the effects of anticancer treatment.

**Figure 2:**
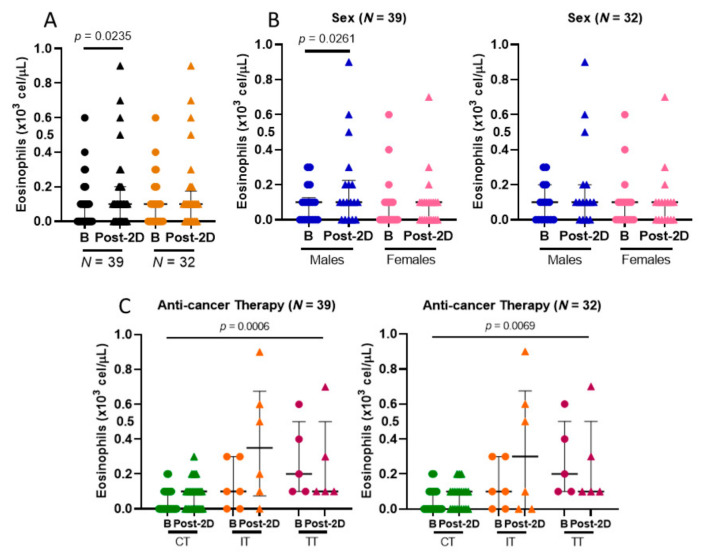
Hematological changes before and after SARS-CoV-2 vaccination. Eosinophil counts were evaluated at Baseline (B) and Post-vaccination (Post-2D) in the complete cohort (*N* = 39) and restricted cohort (*N* = 32). (**A**) Overall eosinophil counts. (**B**,**C**) Eosinophil counts stratified by sex and anticancer therapy. Data are shown as individual values with median and interquartile range. Displayed *p*-values are unadjusted. CT: chemotherapy; IT: immunotherapy; TT: targeted therapy. Additional hematological parameters are shown in Supplementary Figure S1.

Overall, most hematological parameters remained stable between baseline and post-vaccination (Figure S1). Eosinophil counts increased significantly in the complete cohort (*N* = 39; *p* = 0.0235) (Figure 2A). This increase was more evident in males (*p* = 0.0261) and was associated with anticancer therapy, with higher eosinophil counts observed in patients receiving immunotherapy than in those receiving chemotherapy or targeted therapy (*p* = 0.0006) (Figure 2B,C). After excluding the seven patients with detectable baseline anti-SARS-CoV-2 IgG and/or neutralizing antibody activity, the overall increase in eosinophil counts no longer reached statistical significance, whereas the association with anticancer therapy remained significant (*p* = 0.0069) (Figure 2C).

**Figure 3:**
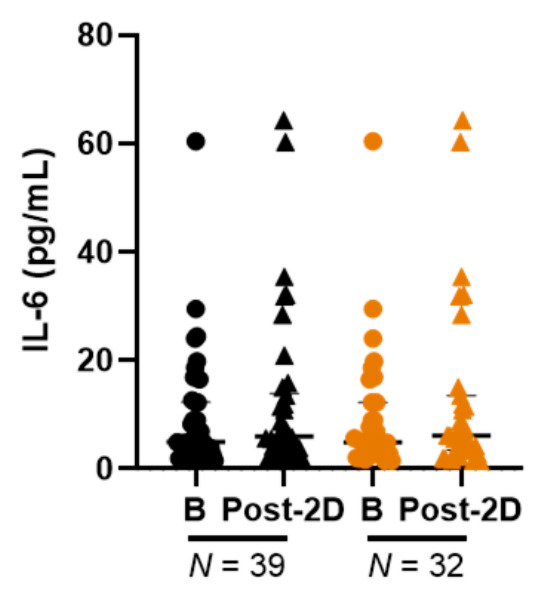
Serum interleukin-6 (IL-6) levels before and after SARS-CoV-2 vaccination. IL-6 levels were evaluated at Baseline (B) and Post-vaccination (Post-2D) in the complete cohort (*N* = 39) and restricted cohort (*N* = 32). Data are shown as individual values with median and interquartile range. Paired comparisons were performed using the Wilcoxon signed-rank test.

Serum IL-6 levels did not change significantly between baseline and post-vaccination, indicating an overall stable inflammatory profile during the study period (Figure 3), although lower IL-6 levels were associated with a higher frequency of generalized pain (*p* = 0.0209) and injection-site pain (*p* = 0.0092).

No consistent association was observed between IL-6 levels and post-vaccination symptoms. These findings suggest that SARS-CoV-2 mRNA vaccination was not associated with a detectable increase in systemic IL-6-mediated inflammation in this cohort of patients with solid tumors undergoing active anticancer treatment.

### 2.4. Cellular Response to SARS-CoV-2 Vaccination

To assess the impact of vaccination on circulating lymphocyte subsets, we analyzed the relative frequencies of immune cell populations by flow cytometry before (Baseline, B) and after vaccination (Post-2D) (Table 3). Given the potential influence of treatment-related leukopenia on absolute leukocyte counts, analyses were primarily performed using relative frequencies.

Overall, vaccination was associated with modest changes in circulating immune cell populations. In the complete cohort (*N* = 39), a moderate increase in total CD3+ T lymphocytes (*p* = 0.0409, r = 0.33) and CD3+CD4+ helper T cells (*p* = 0.0322, r = 0.34) was observed after vaccination. No significant changes were detected in the overall frequencies of CD3+CD8+ T cells, CD3+CD56+ NKT-like cells, CD3−CD56+ NK cells, or in the αβ and γδ T-cell subpopulations. In contrast, circulating CD19+ B-cell frequencies decreased significantly following vaccination (*p* = 0.0138, r = −0.40).

To evaluate the potential influence of pre-existing SARS-CoV-2 immunity, sensitivity analyses were performed after excluding the seven patients with detectable baseline anti-SARS-CoV-2 IgG and/or neutralizing antibody responses (*N* = 32). In this restricted cohort, the increases in total CD3+ and CD3+CD4+ T cells were attenuated and no longer reached statistical significance. In contrast, the reduction in circulating CD19+ B cells remained significant (*p* = 0.0155, r = −0.43), indicating that this finding was consistent irrespective of baseline serological status. Additionally, a modest increase in γδ CD3+CD8+ T-cell frequencies became apparent in the restricted cohort (*p* = 0.0330, r = 0.38).

Taken together, these findings indicate that SARS-CoV-2 mRNA vaccination induced limited changes in the overall composition of circulating lymphocyte subsets in patients with solid tumors. The most consistent cellular finding was a reduction in circulating CD19+ B-cell frequencies, whereas changes observed in T-cell populations were modest and became less evident after exclusion of participants with evidence of prior SARS-CoV-2 exposure.

### 2.5. Dynamics of Global T-Cell Differentiation Stages

T-cell differentiation stages were evaluated by flow cytometry according to CD45RA and CD62L expression, allowing the identification of naïve, central memory (CM), effector memory (EM), and terminal effector memory RA+ (TEMRA) subsets. These differentiation states provide information on T-cell maturation and antigen experience, ranging from less differentiated populations with high proliferative potential to more differentiated effector and memory subsets. The phenotype of each differentiation stage is detailed in Table S2.

**Figure 4:**
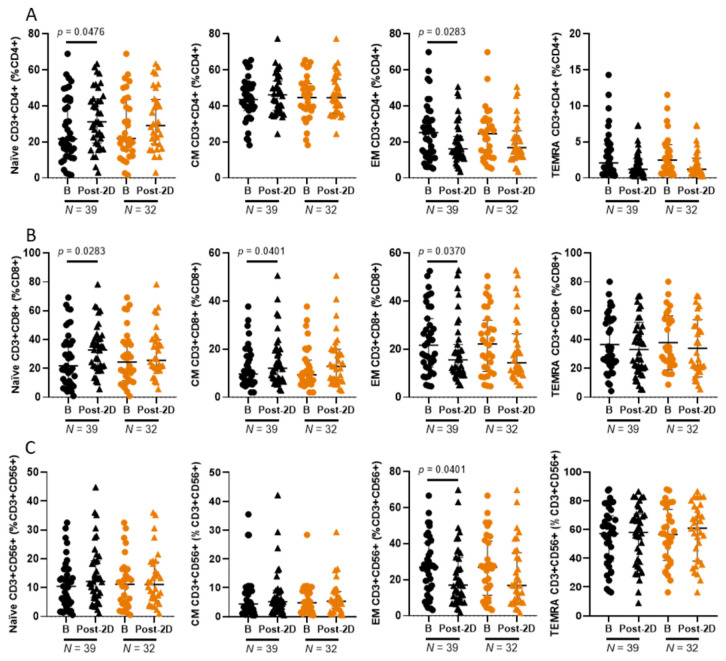
Global T-cell differentiation profiles before and after SARS-CoV-2 vaccination. Relative frequencies of naïve, central memory (CM), effector memory (EM), and terminal effector memory RA+ (TEMRA) subsets within (**A**) CD3+CD4+, (**B**) CD3+CD8+, and (**C**) CD3+CD56+ NKT-like cells at Baseline (B) and after the second vaccine dose (Post-2D) in the complete cohort (*N* = 39) and after exclusion of participants with detectable baseline anti-SARS-CoV-2 IgG and/or neutralizing antibody responses (*N* = 32). Data are shown as individual values with median and interquartile range. Paired comparisons were performed using the Wilcoxon signed-rank test. Adjusted *p*-values correspond to Benjamini–Hochberg false discovery rate (FDR) correction.

Exploratory analyses of T-cell differentiation profiles before and after SARS-CoV-2 vaccination revealed modest changes in the distribution of differentiation subsets (Figure 4). In the complete cohort (*N* = 39), several T-cell populations showed a pattern consistent with increased frequencies of less differentiated subsets and reduced frequencies of more differentiated populations following vaccination.

Within the global CD4+ compartment, naïve T-cell frequencies increased (FDR-adjusted *p* = 0.0476; r = 0.36), whereas effector memory cells decreased (FDR-adjusted *p* = 0.0283; r = −0.46). Similarly, within the global CD8+ compartment, naïve (FDR-adjusted *p* = 0.0283; r = 0.45) and central memory (*p* = 0.0401; r = 0.39) subsets increased, while effector memory CD8+ T cells decreased (FDR-adjusted *p* = 0.0370; r = −0.42). Analyses of absolute cell counts supported these findings, showing a significant increase in naïve CD8+ T cells (FDR-adjusted *p* = 0.0304; r = 0.48) and a trend towards increased central memory CD8+ T-cell counts (Table S3). A similar pattern was observed in CD3+CD56+ NKT-like cells, where effector memory subsets decreased (FDR-adjusted *p* = 0.0401; r = −0.38) and naïve cells showed a non-significant tendency to increase.

To assess the potential influence of pre-existing SARS-CoV-2 immunity, sensitivity analyses were performed after excluding participants with detectable baseline anti-SARS-CoV-2 IgG and/or neutralizing antibody responses (*N* = 32). Under these conditions, the observed differences were attenuated and did not remain statistically significant following false discovery rate (FDR) correction.

Overall, these findings suggest modest changes in T-cell differentiation profiles following SARS-CoV-2 vaccination, characterized by a tendency towards increased frequencies of naïve and central memory subsets and reduced frequencies of effector memory populations. However, the attenuation of these associations after exclusion of participants with evidence of prior SARS-CoV-2 exposure and adjustment for multiple comparisons indicates that these observations should be interpreted cautiously and considered exploratory rather than conclusive.

### 2.6. Differentiation Patterns in αβ and γδ T-Cell Subsets

**Figure 5:**
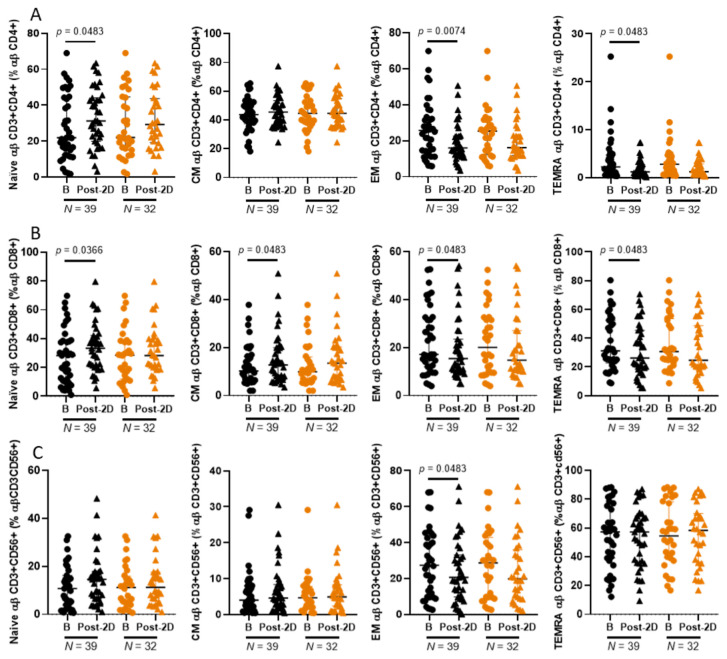
Differentiation patterns of αβ T-cell subsets before and after SARS-CoV-2 vaccination. Relative frequencies of naïve, central memory (CM), effector memory (EM), and terminal effector memory RA+ (TEMRA) subsets within (**A**) αβ CD4+, (**B**) αβ CD8+, and (**C**) αβ CD3+CD56+ NKT-like cells at Baseline (B) and after the second vaccine dose (Post-2D) in the complete cohort (*N* = 39) and after exclusion of participants with detectable baseline anti-SARS-CoV-2 IgG and/or neutralizing antibody responses (*N* = 32). Data are presented as individual values with median and interquartile range. Adjusted *p*-values correspond to Benjamini–Hochberg false discovery rate (FDR) correction.

The differentiation changes observed following SARS-CoV-2 vaccination (Post-2D) in the global T-cell populations were primarily driven by the αβ compartment (Figure 5). In the complete cohort (*N* = 39), αβ CD4+ and αβ CD8+ T cells showed a pattern characterized by increased frequencies of naïve and central memory subsets together with reduced frequencies of effector memory and TEMRA populations after vaccination. Similar directional changes were observed when absolute cell counts were analyzed (Table S3).

In contrast, γδ T-cell subsets showed limited variation following vaccination, and no significant differences were detected in differentiation profiles when relative frequencies were analyzed. However, analyses based on absolute cell counts identified increases in naïve γδ CD8+ and γδ NKT-like cells in the complete cohort.

Following exclusion of participants with detectable baseline anti-SARS-CoV-2 IgG and/or neutralizing antibody responses (*N* = 32), the observed post-vaccination associations were attenuated and did not remain statistically significant after FDR correction. Overall, these findings suggest that the modest differentiation changes observed following SARS-CoV-2 vaccination were mainly attributable to the αβ T-cell compartment, whereas γδ T-cell populations appeared comparatively stable.

### 2.7. Modulation of Apoptosis in Circulating Lymphocyte Subsets

To evaluate the impact of vaccination on lymphocyte survival, early apoptosis was assessed by flow cytometry in the main circulating immune cell populations in both the complete (*N* = 39) and restricted (*N* = 32) cohorts. Early apoptotic cells were defined as Annexin V-positive and 7-aminoactinomycin D (7-AAD)-negative (Table S2, Supplementary Figure S2). As apoptosis is a key regulator of immune cell survival and persistence, changes in this parameter may provide additional insight into cellular immune responses following vaccination.

A marked reduction in early apoptosis was observed following vaccination (Post-2D) across multiple lymphocyte populations (Table 4). In the complete cohort (*N* = 39), apoptosis decreased significantly in total CD3+ T cells (FDR-adjusted *p* = 0.0025; r = −0.51), CD3+CD4+ T cells (FDR-adjusted *p* = 0.0040; r = −0.48), CD3+CD8+ T cells (FDR-adjusted *p* = 0.0012; r = −0.55), CD3−CD56+ NK cells (FDR-adjusted *p* = 0.0097; r = −0.43), and CD3+CD56+ NKT-like cells (FDR-adjusted *p* = 0.0009; r = −0.60). In contrast, no significant changes were detected in CD19+ B-cell apoptosis. Among the major lymphocyte populations, the largest reductions were observed in CD3+CD8+ T cells and CD3+CD56+ NKT-like cells.

Stratification of T cells into αβ and γδ subsets revealed a comparable pattern. Within the αβ compartment, apoptosis decreased significantly in total CD3+ cells, CD3+CD4+ T cells, CD3+CD8+ T cells, and CD3+CD56+ NKT-like cells. Similarly, within the γδ compartment, significant reductions were observed in total CD3+ cells, CD3+CD8+ T cells, CD3+CD56+ NKT-like cells, and double-negative CD3+CD4−CD8− T cells. Multivariate analyses further identified anticancer treatment, particularly chemotherapy, as a factor associated with lower apoptosis rates in γδ CD3+CD8+ T cells (*p* = 0.0154), γδ CD3+CD56+ NKT-like cells (*p* = 0.0079), and double-negative γδ CD3+CD4−CD8− T cells (*p* = 0.0043).

Importantly, the overall apoptosis profile remained highly consistent after exclusion of participants with detectable baseline anti-SARS-CoV-2 IgG and/or neutralizing antibody responses (*N* = 32). The observed reductions persisted after false discovery rate correction, indicating that the findings were not driven by individuals with potential pre-existing SARS-CoV-2 immunity.

Collectively, these results demonstrate a broad reduction in early apoptosis following SARS-CoV-2 vaccination, affecting both conventional and unconventional T-cell populations as well as NK cells. The effect was particularly pronounced in cytotoxic (CD3+CD8+) and innate-like (NKT-like and γδ) lymphocyte compartments, suggesting enhanced cellular survival within immune populations involved in antiviral effector responses.

Correlations between apoptosis rates and post-vaccination anti-SARS-CoV-2 spike-specific IgG levels were assessed using Spearman’s rank correlation analysis. Most lymphocyte populations showed no significant association with humoral vaccine responses. In the overall cohort, modest inverse correlations were observed for selected γδ T-cell subsets, including γδ CD3+CD8+ T cells (rho = −0.3564, *p* = 0.0259), γδ CD3+CD56+ (NKT-like) cells (rho = −0.3929, *p* = 0.0134), and γδ CD3+CD4−CD8− T cells (rho = −0.3289, *p* = 0.0409) (Supplementary Figure S3A). After exclusion of patients with possible prior SARS-CoV-2 exposure, the inverse associations involving γδ CD3+CD8+ T cells (rho = −0.3772, *p* = 0.0333) and γδ CD3+CD56+ (NKT-like) cells (rho = −0.3811, *p* = 0.0314) remained significant, whereas the association observed for γδ CD3+CD4−CD8− T cells was no longer detected (Supplementary Figure S3B).

The consistency of the associations observed for γδ CD3+CD8+ and γδ CD3+CD56+ (NKT-like) cells after exclusion of participants with evidence of prior SARS-CoV-2 exposure suggests that these findings are unlikely to be driven by pre-existing immunity. Overall, these results indicate that apoptosis rates were largely unrelated to vaccine-induced antibody responses, although increased apoptosis within specific γδ T-cell subsets may be associated with lower post-vaccination anti-spike IgG levels. No significant correlations were observed between apoptosis rates and post-vaccination neutralizing activity in either cohort.

### 2.8. Apoptosis Across T-Cell Differentiation Stages

To further characterize the reduction in apoptosis observed after vaccination (Post-2D), early apoptosis was analyzed across T-cell differentiation stages in both the complete (*N* = 39) and restricted (*N* = 32) cohorts, including naïve, central memory (CM), effector memory (EM), and terminal effector memory RA+ (TEMRA) subsets. Overall, vaccination was associated with a broad reduction in apoptosis across multiple differentiation stages, with the most consistent effects observed in CD3+CD8+ T cells and CD3+CD56+ (NKT-like) cells (Table 5).

Within the total T-cell populations, significant reductions in apoptosis were detected across several differentiation stages. The most pronounced and consistent changes occurred in CD3+CD8+ T cells and CD3+CD56+ (NKT-like) cells, where apoptosis decreased across naïve, CM, EM, and TEMRA compartments. CD3+CD4+ T cells also showed reduced apoptosis, particularly within the CM and EM subsets, whereas changes in naïve and TEMRA compartments were less prominent.

A similar pattern was observed in αβ T-cell subsets. Apoptosis was consistently reduced across all differentiation stages of αβ CD3+CD8+ T cells and αβ CD3+CD56+ (NKT-like) cells, while αβ CD3+CD4+ T cells exhibited significant reductions mainly within the CM and EM compartments. Notably, sensitivity analyses performed after exclusion of the seven patients with detectable baseline anti-SARS-CoV-2 IgG and/or neutralizing antibody responses yielded largely comparable results following FDR correction. In this restricted cohort, additional reductions in apoptosis reached statistical significance in naïve and TEMRA CD3+CD4+ T-cell subsets, supporting the robustness of the overall findings.

In contrast, γδ T-cell subsets displayed a less homogeneous response. Significant reductions in apoptosis were primarily observed in γδ CD3+CD56+ (NKT-like) cells, particularly within the EM and TEMRA compartments. Additional decreases were detected in γδ TEMRA CD3+CD8+ T cells and γδ TEMRA CD3+CD4−CD8− T cells, although most γδ differentiation subsets did not exhibit significant changes. Overall, the magnitude and consistency of the apoptotic response were lower in γδ than in αβ T-cell populations.

Taken together, these findings indicate that the reduction in apoptosis following SARS-CoV-2 mRNA vaccination (Post-2D) was not restricted to bulk lymphocyte populations but extended across multiple stages of T-cell differentiation. The most consistent effects were observed within cytotoxic (CD3+CD8+) and innate-like (CD3+CD56+) lymphocyte compartments, particularly among αβ T-cell subsets. Importantly, the persistence of these findings after exclusion of participants with evidence of prior SARS-CoV-2 exposure suggests that the observed reductions in apoptosis were primarily attributable to vaccination rather than pre-existing immunity.

### 2.9. COVID-19 During Follow-Up

Patients were followed for up to 6 months after vaccination, until administration of the third vaccine dose commenced. During follow-up, only one patient (2.56%) developed a confirmed asymptomatic SARS-CoV-2 infection. The affected individual was a male with stage IV lung cancer receiving immunotherapy and without known comorbidities. Post-vaccination anti-SARS-CoV-2 IgG levels in this patient were 139.87 BAU/mL, while neutralizing activity reached 76.07%.

Given the occurrence of only a single breakthrough infection, no formal analyses could be performed to identify immunological correlates of protection. Therefore, no conclusions can be drawn regarding the relationship between humoral immune responses and the risk of SARS-CoV-2 infection in this cohort.

## 3. Discussion

In this prospective explorative observational study, we evaluated humoral and cellular immune responses approximately two months after the second dose of SARS-CoV-2 mRNA vaccination in patients with solid tumors receiving active anticancer treatment, a population that was largely excluded from pivotal vaccine trials despite its increased vulnerability to severe COVID-19 [7,11,31]. While several studies have described vaccine-induced serological responses in oncology patients, information regarding lymphocyte differentiation dynamics and apoptosis remains comparatively limited [12,32].

Our findings demonstrate that mRNA vaccination was associated with robust humoral immunity together with a reduction in early apoptosis across multiple lymphocyte populations and differentiation stages. In contrast, changes in lymphocyte composition and T-cell differentiation were less consistent and appeared more susceptible to the influence of possible prior SARS-CoV-2 exposure. Overall, apoptosis-related findings represented the most robust cellular immune signal identified in this study.

### 3.1. Humoral Response

The humoral response observed in our cohort was consistent with previous reports in patients with solid tumors receiving active treatment. Median post-vaccination anti-spike IgG levels exceeded the 300 BAU/mL threshold proposed in the VOICE study to distinguish adequate from suboptimal responders [19], and both seropositivity (97.44%) and seroconversion rates (96.88%) were comparable to those reported in recent meta-analyses [16,19,20,33].

The similarity of antibody responses observed in both the complete and restricted cohorts suggests that the small subset of patients with baseline seroreactivity did not substantially influence the overall vaccine-induced humoral response. Likewise, no significant associations were detected between antibody levels and demographic or clinical variables, although the moderate sample size and clinical heterogeneity may have limited statistical power.

These findings are in agreement with recent studies demonstrating durable humoral immunity following mRNA vaccination in patients with solid malignancies [20,33]. The low incidence of breakthrough infection observed during follow-up further supports the effectiveness of vaccination in this population, although the limited number of events precluded assessment of immune correlates of protection.

### 3.2. Hematological and Inflammatory Profile

Most hematological and inflammatory parameters remained stable throughout the study period. The main exception was a modest increase in eosinophil counts, particularly among patients receiving immunotherapy. Although eosinophilia has previously been associated with immune activation and immune checkpoint inhibitor treatment [34,35,36,37], clinically relevant eosinophilia was uncommon in our cohort, and its biological significance remains uncertain.

Serum IL-6 levels did not significantly change following vaccination, suggesting the absence of sustained systemic inflammation at the sampling time point evaluated. Because samples were obtained approximately two months after vaccination, transient cytokine responses occurring shortly after vaccine administration may have been missed [38,39]. Overall, these findings indicate that vaccination was not associated with major long-term hematological or inflammatory alterations.

### 3.3. Cellular Immune Changes and T-Cell Differentiation

At the cellular level, vaccination was associated with modest changes in lymphocyte composition and differentiation. Increases in CD3+ and CD3+CD4+ T-cell frequencies and reductions in circulating B cells were observed after vaccination. This reduction may reflect their redistribution to germinal centers or lymphoid tissues, as observed in other studies [40,41,42]. However, most T-cell-related findings were attenuated after exclusion of patients with possible prior SARS-CoV-2 exposure.

Differentiation analyses suggested a relative enrichment of naïve and central memory populations together with reductions in effector memory subsets, particularly within αβ T-cell compartments. Similar patterns have been reported following vaccination and may reflect homeostatic regulation, redistribution of antigen-experienced cells, or memory formation processes [32,43,44,45]. Nevertheless, because these findings were not consistently retained after FDR correction and antigen-specific T-cell responses were not assessed, they should be considered exploratory.

Changes in γδ T-cell differentiation were comparatively limited. Given the indirect mechanisms through which γδ T cells participate in antiviral immune responses and their recognized roles in tumor surveillance and immune regulation [30,46,47], further studies are required to clarify their contribution to vaccine-induced immunity in oncology patients.

Overall, cellular differentiation changes appeared less robust than humoral responses and were more sensitive to possible confounding by prior SARS-CoV-2 exposure and limited sample size.

### 3.4. Apoptosis

The most novel and consistent finding of this study was the reduction in early apoptosis following vaccination. Significant decreases were observed across multiple lymphocyte populations and differentiation stages, particularly among CD3+CD8+ T cells and CD3+CD56+ (NKT-like) cells. Importantly, these findings remained evident after exclusion of patients with possible prior SARS-CoV-2 exposure, supporting the robustness of this finding.

These results are biologically plausible in the context of memory T-cell development. Long-lived memory T cells exhibit increased resistance to apoptosis through modulation of pro- and anti-apoptotic pathways, including increased expression of Bcl-2 family proteins and reduced susceptibility to death receptor-mediated signaling [24,25,26,48]. Preservation of lymphocyte survival during the contraction phase has been linked to the establishment of durable immunological memory [26,49].

The observed reduction in apoptosis may reflect enhanced survival of vaccine-responsive lymphocyte populations and could contribute to the maintenance of adaptive immune responses after vaccination. Although the present study was not designed to assess memory formation directly, these findings are consistent with the concept that successful vaccine-induced immunity requires not only lymphocyte activation but also persistence of antigen-experienced immune cells.

Alternative explanations should nevertheless be considered. Treatment-related effects may contribute to the enrichment of apoptosis-resistant lymphocyte populations, and transient pro-apoptotic signals have been described shortly after mRNA vaccination [31]. Because samples were obtained approximately two months after vaccination, the present study likely reflects a later phase of the immune response, after resolution of acute vaccine-induced inflammatory events.

Our findings complement previous observations in healthy volunteers analyzed using the same methodological approach, where apoptosis dynamics were associated with SARS-CoV-2 exposure history and clinical outcomes [30]. In contrast to that study, reduced apoptosis remained evident after exclusion of individuals with possible prior infection, suggesting that vaccination-related modulation of lymphocyte survival in patients with cancer may occur independently of pre-existing SARS-CoV-2 immunity.

Although the functional significance of these findings remains to be fully established, apoptosis-related changes constituted the most reproducible cellular immune feature identified in this study and may represent a previously underexplored aspect of vaccine-induced immune remodeling in oncology patients.

Notably, apoptosis-related changes remained significant after exclusion of patients with possible prior SARS-CoV-2 exposure and correction for multiple testing, whereas most differentiation-related findings did not. This observation highlights reduced lymphocyte apoptosis as the most robust cellular immune feature identified in the present study.

### 3.5. Implications for mRNA Vaccine Platforms

The expanding use of mRNA technologies for infectious diseases and cancer immunotherapy highlights the importance of understanding vaccine-induced immune remodeling in vulnerable populations [9,10]. Our findings indicate that patients with solid tumors receiving active treatment are capable of mounting robust humoral responses while simultaneously exhibiting changes in lymphocyte survival dynamics. These observations suggest that the immunological impact of mRNA vaccination extends beyond antibody production and may involve broader mechanisms regulating immune homeostasis [50].

### 3.6. Strengths and Limitations

This study has several noteworthy strengths, including its prospective longitudinal design, the integrated assessment of humoral, inflammatory, and cellular immune responses, and the detailed characterization of lymphocyte populations through immunophenotyping, differentiation-stage analysis, and apoptosis profiling. To our knowledge, this is one of the few studies evaluating vaccine-associated changes in apoptosis across both αβ and γδ T-cell subsets in patients with solid tumors receiving active anticancer treatment. Furthermore, the consistency of the apoptosis-related findings across sensitivity analyses strengthens the robustness of the main observations.

Several limitations should nevertheless be considered. The study was conducted in a relatively small and clinically heterogeneous cohort, reflecting the complexity of real-world oncology populations. Patient recruitment was particularly challenging during this phase of the COVID-19 pandemic, as vaccination of vulnerable populations was rapidly prioritized and many patients were understandably reluctant to participate in additional research procedures during a period of heightened concern regarding SARS-CoV-2 infection. Consequently, the available recruitment window was limited, and cohort expansion was difficult.

In addition, antigen-specific SARS-CoV-2 T-cell responses were not directly assessed. This study was conducted during 2021, when standardized and widely accessible assays for measuring SARS-CoV-2-specific cellular immunity were only beginning to emerge and were not routinely available in most clinical research settings. At the same time, vaccination campaigns in oncology patients were implemented rapidly because of the high risk of severe COVID-19 in this population. Immune analyses were therefore performed in real time using the methodologies available at that moment, prioritizing preservation of viable cellular populations and longitudinal immune monitoring during the vaccination process.

Although a contemporaneous healthy control group was not available, the use of a previously characterized healthy cohort analyzed using the same methodological approach provides a valuable contextual framework for interpreting the findings [30]. Finally, the limited number of breakthrough infections prevented exploration of clinical correlates of protection.

Despite these limitations, the study provides novel insights into the immunological effects of SARS-CoV-2 mRNA vaccination in patients with solid tumors, particularly regarding lymphocyte apoptosis dynamics, and establishes a foundation for future mechanistic and translational studies in larger cohorts.

## 4. Materials and Methods

### 4.1. Study Design and Population

**Figure 6:**
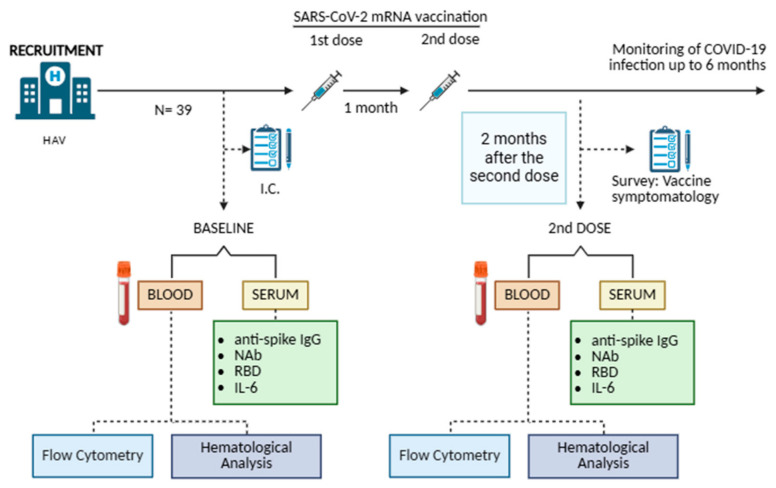
Study design and sample collection timeline. Patients with solid tumors receiving active anticancer treatment were recruited at Hospital Arnau de Vilanova (HAV), Valencia, Spain. Peripheral blood and serum samples were collected at baseline, before the first dose of SARS-CoV-2 mRNA vaccination, and approximately 2 months after the second dose, prior to the next treatment cycle. Blood samples were used for flow cytometry and hematological analysis, whereas serum samples were used to quantify anti-spike IgG, neutralizing antibody response, circulating serum receptor-binding (RBD) levels and interleukin 6 (IL-6). Vaccination-related symptoms were recorded using a standardized questionnaire, and participants were monitored for SARS-CoV-2 infection for up to 6 months. Abbreviations: HAV: Arnau de Vilanova Hospital; I.C: informed consent; Ig: immunoglobulin; NAb: Neutralizing antibodies; RBD: receptor-binding domain; IL: Interleukin; COVID-19: Coronavirus Disease 2019. (Created with BioRender.com).

This was a single-center prospective observational study conducted at Hospital Arnau de Vilanova, Valencia, Spain (HAV). Between April 2021 and November 2021, 39 patients with documented solid tumors receiving active anticancer treatment at the Department of Medical Oncology were recruited. No formal sample size calculation was performed; this was an exploratory hypothesis-generating study, and the final sample size was determined by the number of eligible patients who could be recruited during the study period. A participant flow diagram is provided in Figure 6. The limited sample size reflects the recruitment difficulties encountered during that stage of the pandemic, when the rapid rollout of COVID-19 vaccination among oncology patients restricted the availability of eligible unvaccinated individuals.

All participants received one of the approved SARS-CoV-2 mRNA vaccines. Patients were excluded if they had any additional immunosuppressive condition unrelated to cancer or its treatment, if they had received any other vaccine within the previous 6 months, or if they had a known history of symptomatic COVID-19. A prior history of SARS-CoV-2 infection was specifically assessed at enrollment by clinical interview. Thus, the study was designed to include patients without a known previous history of COVID-19. However, baseline serological analyses indicated that a small number of patients had likely experienced prior mild or asymptomatic SARS-CoV-2 infection despite the absence of reported COVID-19-related symptoms.

Demographic and clinical data were collected for all participants. Vaccination-related symptoms, including cough, fever, dyspnea, sore throat, vomiting, diarrhea, headache, asthenia, anosmia, pain, symptom severity and duration, need for hospital admission or medical appointment, and medication intake, were recorded using a standardized questionnaire. Peripheral blood samples were collected before administration of the first vaccine dose (Baseline) and approximately 2 months after the second dose. To minimize variability related to treatment timing, blood samples were collected on the day of the patients’ scheduled treatment administration, coinciding with routine pre-treatment clinical laboratory testing. Most patients were receiving weekly treatment schedules, and samples were obtained immediately before treatment administration. In patients receiving combined chemotherapy and immunotherapy, for whom immune checkpoint inhibitors (ICIs) were administered every 21 days, blood collection was performed on the scheduled day of ICI administration and prior to infusion. Therefore, all post-vaccination samples were obtained immediately before the next planned treatment cycle.

Hematological parameters, anti-SARS-CoV-2 spike IgG, neutralizing antibody response, serum RBD, IL-6 and lymphocyte subpopulations, including their differentiation stages and apoptosis rates, were analyzed. Baseline immune status was assessed through routine hematological parameters, including leukocyte, lymphocyte, granulocyte, monocyte, eosinophil, basophil and platelet counts, as well as hemoglobin levels. In addition, systemic inflammatory status before and after vaccination was evaluated by measuring circulating IL-6 concentrations, a cytokine closely associated with cancer-related inflammation, immune regulation and treatment-related immune effects [51,52]. Together with the detailed characterization of lymphocyte subsets, differentiation stages and apoptosis profiles, these analyses provided a comprehensive evaluation of the immune landscape and immunological status of cancer patients before and after mRNA vaccination, extending beyond conventional serological assessment of vaccine responses.

Participants were also followed for up to 6 months for the occurrence of SARS-CoV-2 infection. The overall study design is shown in Figure 6.

### 4.2. Ethical Considerations

The study was approved by the Research Ethics Committee of Hospital Arnau de Vilanova, Valencia, Spain (Ethics Committee No. HAV-BAR-2021-03), and was conducted in accordance with the Declaration of Helsinki, the recommendations of the Spanish Bioethics Committee, and Spanish legislation on biomedical research (Law 14/2007). Written informed consent was obtained from all participants before enrollment. Participant privacy and data confidentiality were guaranteed in accordance with Spanish Law 3/2018 and European Regulation (EU) 2016/679.

### 4.3. Blood Sample Processing and Laboratory Analyses

#### 4.3.1. Hematological Analysis

Blood cell counts were measured using an LH750 hematology analyzer (Beckman Coulter, Inc., Brea, CA, USA) as part of routine clinical practice at Hospital Arnau de Vilanova.

#### 4.3.2. Flow Cytometric Immunophenotyping

Blood samples were processed within 2 h of collection. Peripheral blood mononuclear cells (PBMCs) were isolated from EDTA-anticoagulated blood by density-gradient centrifugation using Lymphoprep™ (StemCell Technologies, Vancouver, BC, Canada) at 3500 rpm for 20 min. After two washes in phosphate-buffered saline (PBS), cells were resuspended in 200 µL PBS.

Phenotypic characterization of αβ and γδ T-cell subsets was performed according to previously described methodologies [30,53,54,55]. Briefly, PBMCs were incubated in two separate tubes with anti-TCR PAN αβ-PE and anti-TCR PAN γδ-PE antibodies for 10 min at room temperature protected from light, followed by staining with fluorochrome-conjugated monoclonal antibodies (Beckman Coulter, Inc.). After an additional 10 min incubation, 1 mL of prepared VersaLyse “Fix-and-Lyse” reagent (Beckman Coulter, Inc.) was added. The suspension was mixed and incubated for 20 min at room temperature protected from light. Subsequently, 2 mL PBS were added, and samples were centrifuged at 1500 rpm for 5 min. Cell pellets were finally resuspended in 500 µL PBS.

Data acquisition and analysis were performed on a Navios flow cytometer (Beckman Coulter, Inc.) using Kaluza software (Beckman Coulter, Inc., version 2.2), with 100,000 total events acquired per sample. The Navios flow cytometer (Beckman Coulter, Inc.) was equipped with 3 lasers (violet (405 nm), blue (488 nm) and red (638 nm)) and configured with 10 fluorescence detectors. Daily instrument quality control was performed using Flow-Check and Flow-Set fluorospheres (Beckman Coulter, Inc.), allowing the adjustment of SS, FS and the different lasers. The phenotypic definition of immune cell populations analyzed as well as the full flow cytometry panel detailing antibody clones and fluorochromes, are described in Table S2 and Table S4, respectively. Compensation was performed using VersaComp Antibody Capture Beads (Beckman Coulter, Inc.) stained individually with each fluorochrome-conjugated antibody included in the panel, and automatic compensation matrix was generated using Kaluza software (version 2.2) before sample acquisition. Representative compensation settings are shown in Supplementary Figure S4. The gating strategy used to identify lymphocyte populations is shown in Supplementary Figure S5 (αβ T cells, B cells, and conventional NK cells) and Supplementary Figure S6 (γδ T cells). The absolute counts of circulating cell subsets were calculated using dual-platform counting technology.

Because patients undergoing active anticancer treatment may present treatment-related leukopenia or lymphopenia, flow cytometric data were primarily expressed as relative frequencies (%) of each lymphocyte subset. This approach was considered more representative of changes in immune cell composition during the vaccination period than absolute cell counts alone.

#### 4.3.3. Flow Cytometric Apoptosis Analysis

Apoptosis was assessed using the Annexin V-FITC/7-AAD Kit (Beckman Coulter, Inc.), based on Annexin V binding to phosphatidylserine exposed on the outer leaflet of the plasma membrane during early apoptosis and 7-amino-actinomycin D (7-AAD) binding to DNA. Staining was performed according to the manufacturer’s instructions. To establish the positivity threshold for Annexin V staining, apoptosis was experimentally induced in lymphocytes using hydrogen peroxide (H_2_O_2_) treatment at different concentrations (25 mM and 50 mM) for 15 min. The increase in Annexin V-positive cells observed after H_2_O_2_ exposure was used to define the positive Annexin V gate (Supplementary Figure S7). FMO controls were not performed. For the Annexin V/7-AAD assay, positivity thresholds were established using a combination of unstained controls and the positive-control approach shown in Supplementary Figure S7, and the same gating strategy was consistently applied across all study time points. Gating strategies for apoptosis analyses in the main lymphocyte populations, including αβ and γδ T cells, are shown in Supplementary Figure S2, while unstained apoptosis controls are presented in Supplementary Figure S8. The results described in this work refer to early apoptotic cells, defined as Annexin V-positive and 7-AAD-negative (Annexin V+/7-AAD−).

#### 4.3.4. Serum Antibody and Biomarker Analyses

Anti-SARS-CoV-2 anti-spike IgG antibodies were quantified in serum using the SARS-CoV-2 IgG II Quant assay, a chemiluminescent microparticle immunoassay (CMIA) performed on the ARCHITECT i2000SR platform (Abbott Diagnostics, Mississauga, ON, Canada), according to the manufacturer’s instructions. Results were reported as binding antibody units per milliliter (BAU/mL), standardized against the WHO First International Standard for anti-SARS-CoV-2 immunoglobulin (NIBSC code 20/136). The analytical positivity threshold for this assay was defined as ≥7.10 BAU/mL, with samples below this value reported as negative. In this study, seroconversion was defined as the transition from a seronegative result (<7.10 BAU/mL) at baseline to a seropositive result (≥7.10 BAU/mL) post-vaccination (Post-2D). The upper limit of quantification was 5680 BAU/mL; samples exceeding this value were diluted 1:2 with PBS and re-measured, as recommended by the manufacturer. The intra-assay coefficient of variation was 7%.

The neutralizing antibody response against the SARS-CoV-2 spike receptor-binding domain (RBD) was assessed in serum using the Human SARS-CoV-2 Neutralizing Ab ELISA kit (Thermo Fisher Scientific, Waltham, MA, USA), according to the manufacturer’s instructions. This assay employs a competitive ELISA format in which patient antibodies compete with a labeled ACE2 protein for binding to immobilized RBD, thereby providing a surrogate measure of neutralizing activity based on inhibition of the RBD–ACE2 interaction. It does not assess neutralization of live or pseudotyped viruses. Results were expressed as neutralization rate (%), and the positivity threshold was defined as ≥20%, in accordance with the manufacturer’s specifications. The intra-assay coefficient of variation was 1.40%.

In parallel, circulating serum RBD levels were measured using the Human SARS-CoV-2 RBD ELISA kit (Thermo Fisher Scientific), a sandwich ELISA designed to quantify soluble SARS-CoV-2 spike RBD protein in human serum. The lower limit of detection for this assay was 4.50 pg/mL. The intra-assay coefficient of variation was 10%.

Absorbance for all ELISA assays was measured at 450 nm using a MobiTM microplate reader (MicroDigital, Seongnam-si, Gyeonggi-do, Republic of Korea).

Serum IL-6 levels were determined using the Elecsys immunoassay on a cobas e801 analytical unit (Roche Diagnostics, Basel, Switzerland). The cutoff value for this assay was 1.50 pg/mL, and the maximum value 5000 pg/mL. Intra-assay coefficient of variation was <4.10%.

### 4.4. Statistical Analyses

Descriptive analyses were performed for all study variables. Categorical variables were summarized as absolute and relative frequencies, and continuous variables were expressed as medians and interquartile ranges (IQR), given the small sample size and non-normal distribution of most variables.

Paired comparisons between baseline and post-vaccination values for continuous outcomes were assessed using the Wilcoxon signed-rank test. The standardized Wilcoxon Z statistic was calculated and reported as a measure of effect magnitude. In addition, the effect size (r = (Z/√N) was calculated according to the approach described by Field [56]. Effect sizes were reported for statistically significant comparisons and interpreted as small (r = 0.1), moderate (r = 0.3), and large (r = 0.5). Comparisons between independent groups (e.g., high vs. low responders) were performed using the Mann–Whitney U test. Correlations between continuous variables were evaluated using Spearman’s rank correlation coefficient.

To evaluate the influence of categorical clinical variables on continuous outcomes over time, separate linear mixed-effects models were fitted for each outcome and each clinical covariate of interest (sex, cancer type, disease stage, anticancer therapy, and number of comorbidities) by restricted maximum likelihood (REML) using the lme4 package (version 1.1-35.5) in R. In each model, time point (baseline vs. post-vaccination) was included as a within-subject fixed effect, and the patient was modeled as a random intercept to account for repeated measurements within individuals. Interaction terms between time point and each clinical covariate were included to assess whether changes over time differed across subgroups. Degrees of freedom and *p*-values for fixed effects were estimated using Satterthwaite’s approximation, as implemented in the lmerTest package (version 3.1-3). This approach was selected because it provides acceptable Type I error rates even for smaller sample sizes when models are fitted using REML [57].

To account for multiple comparisons arising from the large number of paired analyses, *p*-values obtained from Wilcoxon signed-rank tests were adjusted using the Benjamini–Hochberg false discovery rate (FDR) correction [58]. FDR correction was applied separately within predefined families of biologically related comparisons, which were defined a priori according to distinct analytical domains. Specifically, corrections were performed independently for: (i) T-cell differentiation stage analyses, (ii) early apoptosis among the main circulating immune populations, and (iii) early apoptosis across T-cell differentiation stage analyses. Mixed-effects models, correlation analyses and other individual analyses were not subjected to FDR correction. A two-sided FDR-adjusted *p*-value < 0.05 was considered statistically significant.

Statistical analyses were performed using R statistical software (version 4.4.2; R Foundation for Statistical Computing, Vienna, Austria). The following R packages were used: lme4 (version 1.1-35.5) and lmerTest (version 3.1-3) for linear mixed-effects models, stats (base R) for Wilcoxon signed-rank and Mann–Whitney U tests, and p.adjust (base R) for Benjamini–Hochberg FDR correction. Figures were generated and statistical analyses were performed using GraphPad Prism (version 8.1.0; GraphPad Software, Boston, MA, USA) for Windows.

## 5. Conclusions

SARS-CoV-2 mRNA vaccination elicited robust humoral immune responses in patients with solid tumors receiving active anticancer treatment, resulting in high seroconversion rates and strong neutralizing antibody activity. These findings confirm the ability of patients with solid malignancies to develop effective vaccine-induced immunity despite ongoing systemic therapy.

Beyond antibody responses, vaccination was associated with a consistent reduction in early apoptosis across multiple lymphocyte populations, particularly CD8+ T cells and CD3+CD56+ (NKT-like) cells. Notably, these findings remained significant after exclusion of patients with possible prior SARS-CoV-2 exposure and correction for multiple testing, identifying reduced lymphocyte apoptosis as the most robust cellular immune feature observed in this study.

Collectively, these results suggest that the immunological effects of mRNA vaccination extend beyond humoral immunity and may involve modulation of lymphocyte survival and immune homeostasis. Further studies are warranted to determine the biological significance of these observations and their potential implications for future mRNA-based vaccination and immunotherapeutic strategies in oncology.

## Figures and Tables

**Table 1:** Baseline clinical and demographic characteristics of study population.

Variable	*N* = 39
Age (median, range)	58 (29–76)
**Sex**	
Male	18 (46.15%)
Female	21 (53.85%)
**Tumor**	
Breast and ovarian	18 (46.15%)
Gastrointestinal	9 (23.08%)
Respiratory	7 (17.95%)
Genitourinary	5 (12.82%)
**Tumor Stage**	
I	3 (7.69%)
II	12 (30.77%)
III	13 (33.33%)
IV	11 (28.21%)
**Type of anti-cancer therapy**	
Chemotherapy	28 (71.79%)
Immunotherapy ^a^	6 (15.39%)
Targeted Therapy ^b^	5 (12.82%)
**Presence of comorbidity**	
Hypertension	12 (30.77%)
Dyslipidemia	6 (15.39%)
Diabetes Mellitus	5 (12.82%)
Hypothyroidism	2 (5.13%)
COPD ^c^	2 (5.13%)
Other ^d^	15 (38.46%)
**Baseline SARS-CoV-2 humoral immunity status ^e^**	
IgG−/NAb−	32 (82.05%)
IgG+/NAb+	5 (12.82%)
IgG−/NAb+	2 (5.13%)

Footnotes: ^a^ Immune Checkpoint Inhibitors (ICIs), ^b^ Hormone therapy, Epidermal Growth Factor Receptor (EGFR) blockade, Human Epidermal Growth Factor Receptor 2 (HER-2) blockade, cyclin kinase inhibitors (CDKis), antiangiogenic drugs, ^c^ Chronic Obstructive Pulmonary Disease, ^d^ Other Comorbidities include: Cervical osteoarthritis, Hyperthyroidism, Cholelithiasis, Chronic Migraines, Wolff-Parkinson-White Syndrome, Chronic Kidney Failure, Gastritis, Bronchopathy, Vitiligo, Polycystic Ovary Syndrome. ^e^ IgG: anti-SARS-CoV-2 spike immunoglobulin G; NAb: neutralizing antibodies against SARS-CoV-2.

**Table 2:** Pairwise comparisons (Wilcoxon test) of humoral immunity parameters between samples obtained at Baseline (B) and Post-vaccination (Post-2D).

	*N* = 39				*N* = 32			
	B Median (IQR)	Post-2D Median (IQR)	Wilcoxon Z	*p*-Value	B Median (IQR)	Post-2D Median (IQR)	Wilcoxon Z	*p*-Value
Anti-spike IgG (BAU/mL)	0 (0–0)	988.69 (152.22–3798.05)	5.3731	<0.0001	0 (0–0)	1082.71 (285.16–4073.29)	4.8599	<0.0001
Neutralizing antibody response (%)	0 (0–0)	85.73 (70.42–92.34)	5.4145	<0.0001	0 (0–0)	86.06 (70.60–92.42)	4.9365	<0.0001

Footnotes: BAU: Binding antibody units; IgG: Immunoglobulin G; IQR: Interquartile range.

**Table 3:** Pairwise comparisons (Wilcoxon test) of immune cell populations between Baseline (B) and Post-vaccination (Post-2D) time points in the complete (*N* = 39) and restricted cohorts (*N* = 32).

			*N* = 39			*N* = 32			
Cell Population (% Total Lymphocytes)		B Median (IQR)	Post-2D Median (IQR)	Wilcoxon Z	*p*-Value	B Median (IQR)	Post-2D Median (IQR)	Wilcoxon Z	*p*-Value
CD3+	Total	67.08 (50.78–80.01)	69.56 (62.94–79.55)	2.0514	**0.0409**	68.60 (52.30–80.50)	70.31 (62.81–79.61)	1.5707	0.1184
	αβ	62.21 (48.47–73.91)	64.26 (57.86–75.12)	1.6467	0.1011	63.62 (49.10–76.66)	64.84 (56.62–75.85)	1.2154	0.2278
	γδ	0.91 (0.51–3.54)	1.35 (0.60–3.92)	0.9768	0.3321	0.82 (0.47–1.59)	1.20 (0.54–2.83)	1.0658	0.2907
CD3+CD4+	Total	40.78 (29–52.63)	45.23 (32.51–55.04)	2.1491	**0.0322**	39.62 (27.80–52.64)	44.56 (29.57–53.49)	1.6268	0.1058
	αβ	40.78 (28.91–53.18)	45.51 (33.99–62.59)	1.9118	0.0568	42.60 (27.77–53.13)	47.35 (31.86–61.38)	1.7016	0.0906
	γδ ^a^	ND	ND	ND	ND	ND	ND	ND	ND
CD3+CD8+	Total	19.77 (12.83–29.48)	21.57 (17.35–32.34)	1.5909	0.1132	19.00 (15.47–27.68)	24.17 (17.36–33.00)	1.5894	0.1141
	αβ	18.51 (12.69–25.62)	22.17 (16.39–28.71)	1.5909	0.1132	18.34 (14.46–26.50)	23.90 (16.86–33.26)	1.6268	0.1058
	γδ	0.31 (0.11–1.15)	0.64 (0.18–1.99)	1.9467	0.0524	0.27 (0.11–0.98)	0.46 (0.18–1.29)	2.1410	**0.0330**
CD3+CD56+ (NKT-like)	Total	4.12 (1.89–7.47)	5.88 (1.94–11.24)	1.3118	0.1920	4.12 (2.07–7.76)	5.80 (1.86–9.20)	1.2902	0.2002
	αβ	3.27 (1.49–5.91)	4.21 (1.93–6.56)	1.1024	0.2733	3.85 (1.75–6.19)	4.39 (1.81–6.86)	1.0284	0.3082
	γδ	0.39 (0.12–1.09)	0.66 (0.33–2.12)	1.4106	0.1606	0.36 (0.09–0.61)	0.54 (0.21–2.00)	1.5323	0.1280
CD3+CD4-CD8-	γδ	0.56 (0.28–2.62)	0.89 (0.34–2.70)	1.5211	0.1300	0.54 (0.22–1.10)	0.66 (0.31–1.64)	1.7390	0.0837
CD3-CD56+ (NK)		17.41 (8.32–29.91)	19.16 (12.99–32.08)	−0.0977	0.9277	16.25 (7.15–29.82)	21.84 (13.17–32.28)	0.1683	0.8737
CD19+		5.63 (3.49–9.14)	4.24 (2.86–6.35)	−2.4700	**0.0138**	5.88 (4.39–9.19)	4.20 (3.24–5.91)	−2.4309	**0.0155**

Footnotes: ^a^ CD3+CD4+ γδ T cells were not detected. NK: Natural killer; NKT-like: Natural Killer T-like; IQR: Interquartile range; ND: Not detected.

**Table 4:** Pairwise comparisons (Wilcoxon test) of early apoptosis across lymphocyte subsets between Baseline (B) and Post-vaccination (Post-2D) time points in the complete (*N* = 39) and restricted (*N* = 32) cohorts.

		*N* = 39					*N* = 32				
Cell Population (% Apoptosis)		Baseline Median (IQR)	Post-2D Median (IQR)	Wilcoxon Z	*p*-Value	*p*-Value (FDR)	Baseline Median (IQR)	Post-2D Median (IQR)	Wilcoxon Z	*p*-Value	*p*-Value (FDR)
CD3+	Total	1.42 (0.44–2.22)	0.48 (0.15–1.06)	−3.1957	**0.0014**	**0.0025**	1.50 (0.76–2.18)	0.45 (0.18–1.02)	−3.1975	**0.0014**	**0.0025**
	αβ	1.40 (0.44–2.20)	0.48 (0.15–1.10)	−3.1538	**0.0017**	**0.0026**	1.41 (0.75–2.18)	0.45 (0.18–0.99)	−3.1414	**0.0017**	**0.0028**
	γδ	1.61 (0.58–2.73)	0.41 (0.01–1.11)	−3.5096	**0.0005**	**0.0012**	1.64 (0.74–2.91)	0.39 (0–1.07)	−3.5274	**0.0004**	**0.0011**
CD3+CD4+	Total	0.83 (0.29–1.19)	0.32 (0.09–0.64)	−2.9724	**0.0030**	**0.0040**	0.86 (0.29–1.24)	0.32 (0.11–0.64)	−3.0853	**0.0021**	**0.0028**
	αβ	0.82 (0.29–1.19)	0.32 (0.08–0.64)	−3.0213	**0.0026**	**0.0037**	0.86 (0.29–1.24)	0.32 (0.11–0.64)	−3.1040	**0.0020**	**0.0028**
	γδ ^a^	ND	ND	ND	ND	ND	ND	ND	ND	ND	ND
CD3+CD8+	Total	2.68 (0.82–4.87)	0.70 (0.22–2.23)	−3.4608	**0.0006**	**0.0012**	2.76 (1.03–4.75)	0.68 (0.24–1.78)	−3.5341	**0.0004**	**0.0011**
	αβ	2.78 (0.84–4.88)	0.70 (0.23–2.33)	−3.4329	**0.0006**	**0.0012**	2.81 (1.03–4.80)	0.68 (0.25–1.86)	−3.4967	**0.0005**	**0.0011**
	γδ	1.50 (0–3.00)	0.52 (0–1.12)	−2.6266	**0.0089**	**0.0101**	1.68 (0–3.19)	0.52 (0–1.23)	−2.6668	**0.0079**	**0.0091**
CD3+CD56+ (NKT-like)	Total	2.56 (1.02–5.98)	0.87 (0.26–2.00)	−3.7678	**0.0002**	**0.0009**	2.62 (1.39–5.90)	0.86 (0.31–1.99)	−3.6837	**0.0002**	**0.0010**
	αβ	2.68 (1.04–6.46)	1.00 (0.30–2.36)	−3.8795	**0.0001**	**0.0009**	2.70 (1.37–6.37)	0.99 (0.34–1.88)	−3.8333	**0.0001**	**0.0010**
	γδ	1.69 (0.22–3.68)	0.56 (0–1.00)	−3.5215	**0.0004**	**0.0012**	1.71 (0.15–3.62)	0.50 (0–1.08)	−3.4613	**0.0006**	**0.0011**
CD3+CD4- CD8-	γδ	1.26 (0.22–2.84)	0.36 (0–0.78)	−3.5379	**0.0004**	**0.0012**	1.50 (0.30–2.89)	0.30 (0–0.77)	−3.6662	**0.0003**	**0.0010**
CD3-CD56+ (NK)		0.94 (0.52–2.36)	0.39 (0.12–0.84)	−2.6654	**0.0079**	**0.0097**	1.19 (0.54–2.32)	0.38 (0.13–0.76)	−2.8422	**0.0046**	**0.0057**
CD19+		1.08 (0.59–1.94)	1.46 (0.43–2.17)	0.1523	0.8847	0.9437	1.29 (0.90–2.17)	1.49 (0.42–2.17)	0.5610	0.5812	0.6199

Footnotes: ^a^ CD3+CD4+ γδ T cells were not detected. NK: Natural killer; NKT-like: Natural Killer T-like; IQR: Interquartile range; FDR: False discovery rate; ND: Not detected.

**Table 5:** Pairwise comparisons (Wilcoxon test) of early apoptosis across T-cell differentiation stages between Baseline (B) and Post-vaccination (Post-2D) in the complete (*N* = 39) and restricted (*N* = 32) cohorts.

			*N* = 39					*N* = 32				
T Cell Differentiation Stages (% Apoptosis)			B Median (IQR)	Post-2D Median (IQR)	Wilcoxon Z	*p*-Value	*p*-Value (FDR)	B Median (IQR)	Post-2D Median (IQR)	Wilcoxon Z	*p*-Value	*p*-Value (FDR)
CD3+CD4+	Total	Naïve	0.33 (0.11–0.76)	0.23 (0.07–0.55)	−1.7910	0.0744	0.1010	0.38 (0.14–0.86)	0.23 (0.06–0.43)	−2.5084	**0.0125**	**0.0197**
		CM	0.84 (0.28–1.48)	0.30 (0.08–0.64)	−2.8398	**0.0046**	**0.0096**	0.86 (0.30–1.53)	0.26 (0.10–0.67)	−2.9077	**0.0037**	**0.0089**
		EM	0.92 (0.38–1.53)	0.43 (0.14–0.86)	−3.3492	**0.0008**	**0.0039**	0.96 (0.58–1.54)	0.42 (0.14–0.84)	−3.7024	**0.0002**	**0.0028**
		TEMRA	1.17 (0.44–2.08)	0.66 (0.06–1.10)	−2.0793	**0.0382**	0.0559	1.21 (0.72–2.00)	0.62 (0.14–0.98)	−2.6552	**0.0081**	**0.0155**
	αβ	Naïve	0.32 (0.11–0.64)	0.23 (0.07–0.56)	−1.7258	0.0857	0.1123	0.34 (0.14–0.71)	0.22 (0.06–0.43)	−2.5182	**0.0121**	**0.0197**
		CM	0.84 (0.29–1.44)	0.30 (0.08–0.71)	−2.8747	**0.0041**	**0.0096**	0.86 (0.30–1.53)	0.26 (0.10–0.67)	−2.9544	**0.0032**	**0.0082**
		EM	0.94 (0.39–1.49)	0.43 (0.14–0.86)	−3.3911	**0.0007**	**0.0039**	0.96 (0.58–1.54)	0.43 (0.14–0.84)	−3.7398	**0.0002**	**0.0028**
		TEMRA	1.18 (0.70–2.06)	0.65 (0–1.17)	−2.1351	**0.0333**	0.0507	1.22 (0.76–2.03)	0.61 (0.10–0.95)	−2.8796	**0.0041**	**0.0092**
	γδ ^a^		ND	ND	ND	ND	ND	ND	ND	ND	ND	ND
CD3+CD8+	Total	Naïve	2.15 (0.66–4.93)	0.69 (0.28–2.34)	−2.8497	**0.0045**	**0.0096**	2.27 (0.98–4.50)	0.68 (0.28–1.55)	−3.0375	**0.0025**	**0.0067**
		CM	2.41 (1.16–5.92)	0.71 (0.11–2.14)	−3.5446	**0.0004**	**0.0026**	2.42 (1.31–5.94)	0.71 (0.23–2.14)	−3.2910	**0.0010**	**0.0054**
		EM	3.10 (0.88–6.22)	0.80 (0.18–2.04)	−3.6143	**0.0003**	**0.0026**	3.12 (1.04–5.02)	0.82 (0.24–1.86)	−3.3471	**0.0008**	**0.0054**
		TEMRA	2.30 (0.85–4.70)	0.89 (0.22–2.05)	−3.2376	**0.0012**	**0.0047**	2.28 (0.95–4.66)	0.82 (0.34–1.94)	−3.1227	**0.0018**	**0.0064**
	αβ	Naïve	2.16 (0.67–4.64)	0.67 (0.25–2.47)	−2.9026	**0.0038**	**0.0096**	2.19 (0.86–4.50)	0.66 (0.25–1.52)	−3.0666	**0.0022**	**0.0065**
		CM	2.42 (1.18–5.44)	0.63 (0.11–2.18)	−3.5725	**0.0004**	**0.0026**	2.44 (1.31–5.89)	0.64 (0.24–2.17)	−3.3097	**0.0010**	**0.0054**
		EM	3.01 (0.92–6.02)	0.81 (0.22–2.09)	−3.5655	**0.0004**	**0.0026**	3.08 (1.06–5.08)	0.83 (0.28–1.86)	−3.2630	**0.0011**	**0.0054**
		TEMRA	2.38 (0.86–4.70)	1.00 (0.25–2.26)	−3.2096	**0.0014**	**0.0047**	2.39 (0.99–4.68)	0.88 (0.36–2.06)	−3.1227	**0.0018**	**0.0064**
	γδ	Naïve	0 (0–3.82)	0 (0–2.06)	−1.3143	0.1936	0.2299	0 (0–3.73)	0 (0–2.50)	−1.1268	0.2684	0.3000
		CM ^b^	ND	ND	ND	ND	ND	ND	ND	ND	ND	ND
		EM	0 (0–0.90)	0 (0–0)	−1.2927	0.2052	0.2363	0 (0–1.46)	0 (0–0)	−1.5025	0.1422	0.1689
		TEMRA	1.02 (0–3.06)	0.53 (0–1.52)	−2.2340	**0.0261**	**0.0414**	0.95 (0–2.84)	0.55 (0–1.47)	−1.7893	0.0758	0.0993
CD3+CD56+ (NKT-like)	Total	Naïve	3.30 (1.75–7.96)	1.01 (0.24–3.46)	−3.5585	**0.0004**	**0.0026**	4.24 (2.69–8.24)	0.98 (0.37–3.25)	−3.9829	**0.0001**	**0.0027**
		CM	2.66 (0.33–6.15)	0.57 (0–1.95)	−2.7532	**0.0060**	**0.0115**	3.13 (0.64–6.26)	0.74 (0.18–2.27)	−2.6366	**0.0086**	**0.0156**
		EM	2.69 (0.63–5.88)	0.82 (0.12–1.96)	−2.8259	**0.0048**	**0.0096**	2.88 (1.20–5.62)	0.95 (0.26–2.44)	−2.4589	**0.0143**	**0.0217**
		TEMRA	2.30 (0.90–5.20)	0.67 (0.15–1.68)	−3.6074	**0.0003**	**0.0026**	2.38 (0.98–5.02)	0.62 (0.15–1.62)	−3.5715	**0.0004**	**0.0035**
	αβ	Naïve	3.01 (0.99–6.14)	1.18 (0.06–4.38)	−2.3863	**0.0173**	**0.0300**	3.53 (2.10–7.12)	1.09 (0.01–2.98)	−3.0666	**0.0022**	**0.0065**
		CM	2.78 (0–6.74)	0.70 (0–2.38)	−2.4037	**0.0166**	**0.0300**	2.97 (0.57–6.63)	0.77 (0–2.35)	−2.3935	**0.0171**	**0.0241**
		EM	2.94 (1.13–5.78)	0.71 (0.14–2.04)	−3.0092	**0.0027**	**0.0078**	3.00 (1.45–5.56)	0.79 (0.22–2.23)	−2.5805	**0.0101**	**0.0175**
		TEMRA	2.33 (0.64–4.35)	0.71 (0.15–2.11)	−3.2920	**0.0010**	**0.0043**	2.40 (0.96–4.22)	0.72 (0.16–1.84)	−3.1882	**0.0015**	**0.0062**
	γδ	Naïve	0.51 (0–3.98)	0 (0–1.38)	−2.0191	**0.0488**	0.0631	0.72 (0–4.58)	0 (0–1.91)	−1.8666	0.0646	0.0877
		CM	0 (0–1.12)	0 (0–1.09)	0.3997	0.7022	0.7412	0 (0–1.25)	0 (0–0.98)	0.3077	0.7764	0.8195
		EM	0.36 (0–4.08)	0 (0–0.97)	−2.9732	**0.0031**	**0.0084**	0.64 (0–4.66)	0 (0–0.99)	−2.7285	**0.0067**	**0.0139**
		TEMRA	1.55 (0–5.83)	0 (0–1.17)	−3.0544	**0.0023**	**0.0074**	1.94 (0–5.60)	0 (0–1.24)	−2.7143	**0.0069**	**0.0139**
CD3+CD4- CD8-	γδ	Naïve	0 (0–2.20)	0 (0–0.80)	−1.3383	0.1858	0.2278	0 (0–2.44)	0 (0–0.81)	−1.4154	0.1634	0.1882
		CM	0 (0–1.19)	0 (0–0.68)	−1.2339	0.2238	0.2501	0 (0–1.28)	0 (0–0.67)	−1.7581	0.0832	0.1054
		EM	0.54 (0–2.58)	0 (0–0.76)	−1.5496	0.1241	0.1572	0.69 (0–2.85)	0 (0–0.72)	−1.6233	0.1080	0.1324
		TEMRA	1.28 (0–2.66)	0 (0–0.97)	−2.2728	**0.0237**	**0.0391**	1.59 (0–2.78)	0 (0–0.90)	−2.4000	**0.0170**	**0.0241**

Footnotes: ^a^ CD3+CD4+ γδ T cells were not detected. ^b^ Early apoptosis in central memory γδ CD3+CD8+ T cells was not detected. CM: Central memory; EM: Effector memory; TEMRA: Terminal effector memory RA+; NKT-like: Natural Killer T-like; IQR: Interquartile range; FDR: False discovery rate; ND: Not detected.

## Data Availability

The original contributions presented in this study are included in the article/Supplementary Materials. Further inquiries can be directed to the corresponding author.

## Supplementary Materials

The following supporting information can be downloaded at: https://www.mdpi.com/article/10.3390/ijms27146173/s1.

## Abbreviations

B (Baseline); Post-2D (Post-vaccination); mRNA (Messenger RNA); LNP (Lipid nanoparticle); SARS-CoV-2 (Severe acute respiratory syndrome coronavirus 2); BAU (Binding antibody units); CMIA (Chemiluminescent microparticle immunoassay); ELISA (Enzyme-linked immunosorbent assay); RBD (Receptor-binding domain); ACE2 (human angiotensin-converting enzyme 2); IL (Interleukin); NKT (Natural killer T); NK (Natural killer); CM (Central memory); EM (Effector memory); TEMRA (Terminal effector memory RA+); FDR (False discovery rate); 7-AAD (7-amino-actinomycin D); ICIs (Immune checkpoint inhibitors); PBMCs (Peripheral blood mononuclear cells); PBS (Phosphate-buffered saline); IQR (Interquartile range); REML (Restricted maximum likelihood).

## References

[1] Szabó G.T., Mahiny A.J., Vlatkovic I. (2022). COVID-19 mRNA vaccines: Platforms and current developments. Mol. Ther..

[2] Alameh M.-G., Tombácz I., Bettini E., Lederer K., Ndeupen S., Sittplangkoon C., Wilmore J.R., Gaudette B.T., Soliman O.Y., Pine M. (2021). Lipid nanoparticles enhance the efficacy of mRNA and protein subunit vaccines by inducing robust T follicular helper cell and humoral responses. Immunity.

[3] Verbeke R., Hogan M.J., Loré K., Pardi N. (2022). Innate immune mechanisms of mRNA vaccines. Immunity.

[4] Baden L.R., El Sahly H.M., Essink B., Kotloff K., Frey S., Novak R., Diemert D., Spector S.A., Rouphael N., Creech C.B. (2021). Efficacy and Safety of the mRNA-1273 SARS-CoV-2 Vaccine. N. Engl. J. Med..

[5] Polack F.P., Thomas S.J., Kitchin N., Absalon J., Gurtman A., Lockhart S., Perez J.L., Pérez Marc G., Moreira E.D., Zerbini C. (2020). Safety and efficacy of the BNT162b2 mRNA COVID-19 vaccine. N. Engl. J. Med..

[6] Park J.K., Lee E.B., Winthrop K.L. (2024). What rheumatologists need to know about mRNA vaccines: Current status and future of mRNA vaccines in autoimmune inflammatory rheumatic diseases. Ann. Rheum. Dis..

[7] Li S., Zheng L., Zhong J., Gao X. (2025). Advancing mRNA vaccines for infectious diseases: Key components, innovations, and clinical progress. Essays Biochem..

[8] Whitaker J.A., Sahly H.M.E., Healy C.M. (2023). mRNA vaccines against respiratory viruses. Curr. Opin. Infect. Dis..

[9] Sayour E.J., Boczkowski D., Mitchell D.A., Nair S.K. (2024). Cancer mRNA vaccines: Clinical advances and future opportunities. Nat. Rev. Clin. Oncol..

[10] Li H., Min L., Du H., Wei X., Tong A. (2025). Cancer mRNA vaccines: Clinical application progress and challenges. Cancer Lett..

[11] Lièvre A., Turpin A., Le Malicot K., Thariat J., Ahle G., Neuzillet C., Paoletti X., Aldabbagh K., Michel P., Debieuvre D. (2020). Risk factors for Coronavirus Disease 2019 (COVID-19) severity and mortality among solid cancer patients and impact of the disease on anticancer treatment: A French nationwide cohort study (GCO-002 CACOVID-19). Eur. J. Cancer.

[12] Kamboj M., Bohlke K., Baptiste D.M., Dunleavy K., Fueger A., Jones L., Kelkar A.H., Law L.Y., LeFebvre K.B., Ljungman P. (2024). Vaccination of Adults With Cancer: ASCO Guideline. J. Clin. Oncol..

[13] Desai A., Sachdeva S., Parekh T., Desai R. (2020). COVID-19 and cancer: Lessons from a pooled meta-analysis. JCO Glob. Oncol..

[14] Corti C., Crimini E., Curigliano G. (2021). SARS-CoV-2 vaccines and cancer patients. Ann. Oncol..

[15] Romano E., Pascolo S., Ott P. (2021). Implications of mRNA-based SARS-CoV-2 vaccination for cancer patients. J. Immunother. Cancer.

[16] Becerril-Gaitan A., Vaca-Cartagena B.F., Ferrigno A.S., Mesa-Chavez F., Barrientos-Gutiérrez T., Tagliamento M., Lambertini M., Villarreal-Garza C. (2021). Immunogenicity and risk of Severe Acute Respiratory Syndrome Coronavirus 2 (SARS-CoV-2) infection after Coronavirus Disease 2019 (COVID-19) vaccination in patients with cancer: A systematic review and meta-analysis. Eur. J. Cancer.

[17] Bin Lee A.R.Y., Wong S.Y., Chai L.Y.A., Lee S.C., Lee M.X., Muthiah M.D., Tay S.H., Teo C.B., Tan B.K.J., Chan Y.H. (2022). Efficacy of COVID-19 vaccines in immunocompromised patients: Systematic review and meta-analysis. BMJ.

[18] Yin J., Chen Y., Li Y., Zhang X., Wang C. (2022). Seroconversion rate after COVID-19 vaccination in patients with solid cancer: A systematic review and meta-analysis. Hum. Vaccines Immunother..

[19] Oosting S.F., Van der Veldt A.A.M., GeurtsvanKessel C.H., Fehrmann R.S.N., van Binnendijk R.S., Dingemans A.-M.C., Smit E.F., Hiltermann T.J.N., Hartog G.D., Jalving M. (2021). mRNA-1273 COVID-19 vaccination in patients receiving chemotherapy, immunotherapy, or chemoimmunotherapy for solid tumours: A prospective, multicentre, non-inferiority trial. Lancet Oncol..

[20] Liu A., Necela B., Li Z., Cogen D., Wieczorek M.A., Mummareddy A., Acampora M., Reynolds G.A., Advani P.P., Moreno-Aspitia A. (2026). Long-term cellular and humoral responses to SARS-CoV-2 vaccinations in patients with solid malignancies undergoing chemotherapy. Front. Immunol..

[21] Cortés A., Casado J.L., Longo F., Serrano J.J., Saavedra C., Velasco H., Martin A., Chamorro J., Rosero D., Fernández M. (2022). Limited T cell response to SARS-CoV-2 mRNA vaccine among patients with cancer receiving different cancer treatments. Eur. J. Cancer.

[22] Monin L., Laing A.G., Muñoz-Ruiz M., McKenzie D.R., del Molino del Barrio I., Alaguthurai T., Domingo-Vila C., Hayday T.S., Graham C., Seow J. (2021). Safety and immunogenicity of one versus two doses of the COVID-19 vaccine BNT162b2 for patients with cancer: Interim analysis of a prospective observational study. Lancet Oncol..

[23] Matsumura T., Takano T., Takahashi Y. (2023). Immune responses related to the immunogenicity and reactogenicity of COVID-19 mRNA vaccines. Int. Immunol..

[24] Kurtulus S., Tripathi P., Hildeman D.A. (2013). Protecting and rescuing the effectors: Roles of differentiation and survival in the control of memory T cell development. Front. Immunol..

[25] Chang J.T., Wherry E.J., Goldrath A.W. (2014). Molecular regulation of effector and memory T cell differentiation. Nat. Immunol..

[26] Grayson J.M., Zajac A.J., Altman J.D., Ahmed R. (2000). Cutting edge: Increased expression of Bcl-2 in antigen-specific memory CD8^+^ T cells. J. Immunol..

[27] Mensurado S., Blanco-Domínguez R., Silva-Santos B. (2023). The emerging roles of γδ T cells in cancer immunotherapy. Nat. Rev. Clin. Oncol..

[28] Stevens G., Blanco-Domínguez R., Mensurado S., Silva-Santos B. (2026). γδ T Cells for Cancer Immunotherapy: Unconventional Players Take the Spotlight. Annu. Rev. Immunol..

[29] Garde-Noguera J., Fernández-Murga M.L., Giner-Bosch V., Dominguez-Márquez V., Sánchez J.G., Soler-Cataluña J.J., Chuliá F.L., Honrubia B., Piera N., Llombart-Cussac A. (2020). Impact of SARS-CoV-2 Infection on Patients with Cancer: Retrospective and Transversal Studies in Spanish Population. Cancers.

[30] Andreu-Ballester J.C., Galindo-Regal L., Cuéllar C., López-Chuliá F., García-Ballesteros C., Fernández-Murga L., Llombart-Cussac A., Domínguez-Márquez M.V. (2024). A Low Number of Baselines γδ T Cells Increases the Risk of SARS-CoV-2 Post-Vaccination Infection. Vaccines.

[31] Gimenez S., Hamrouni E., André S., Picard M., Soundaramourty C., Lozano C., Vincent T., Tran T.-A., Kundura L., Estaquier J. (2025). Monocytic reactive oxygen species–induced T-cell apoptosis impairs cellular immune response to SARS-CoV-2 mRNA vaccine. J. Allergy Clin. Immunol..

[32] Pfannes R., Pierzchalski A., Maddalon A., Simion A., Zouboulis C.C., Behre G., Zenclussen A.C., Westphal S., Fest S., Herberth G. (2023). Characterization of post-vaccination SARS-CoV-2 T cell subtypes in patients with different hematologic malignancies and treatments. Front. Immunol..

[33] Favalli A., Patelli G., Gruarin P., Gobbini A., Pesce E., Mariano S., Bombaci M., Vincenti F., Donnici L., Marchese S. (2025). Impact of chemotherapy on humoral and cellular immune responses to COVID-19 vaccination in patients with solid tumors. Front. Immunol..

[34] Rosenberg H.F., Foster P.S. (2021). Eosinophils and COVID-19: Diagnosis, prognosis, and vaccination strategies. Semin. Immunopathol..

[35] Simon S.C.S., Hu X., Panten J., Grees M., Renders S., Thomas D., Weber R., Schulze T.J., Utikal J., Umansky V. (2020). Eosinophil accumulation predicts response to melanoma treatment with immune checkpoint inhibitors. OncoImmunology.

[36] Moore K.M., Foster S.L., Kar M., Floyd K.A., Elrod E.J., Williams M.E., Velden J.V., Ellis M., Malik A., Wali B. (2025). Monocyte-eosinophil signaling axis promotes vaccine-mediated protection against SARS-CoV-2. PLoS Pathog..

[37] Park Y.M., Bochner B.S. (2010). Eosinophil survival and apoptosis in health and disease. Allergy Asthma Immunol. Res..

[38] Spiliopoulou P., van Rensburg H.J.J., Avery L., Kulasingam V., Razak A., Bedard P., Hansen A., Chruscinski A., Wang B., Kulikova M. (2023). Longitudinal efficacy and toxicity of SARS-CoV-2 vaccination in cancer patients treated with immunotherapy. Cell Death Dis..

[39] Walle T., Bajaj S., Kraske J.A., Rösner T., Cussigh C.S., Kälber K.A., Müller L.J., Strobel S.B., Burghaus J., Kallenberger S.M. (2022). Cytokine release syndrome-like serum responses after COVID-19 vaccination are frequent and clinically inapparent under cancer immunotherapy. Nat. Cancer.

[40] Reynolds C.J., Pade C., Gibbons J.M., Butler D.K., Otter A.D., Menacho K., Fontana M., Smit A., Sackville-West J.E., Cutino-Moguel T. (2021). Prior SARS-CoV-2 infection rescues B and T cell responses to variants after first vaccine dose. Science.

[41] Kim W., Zhou J.Q., Horvath S.C., Schmitz A.J., Sturtz A.J., Lei T., Liu Z., Kalaidina E., Thapa M., Alsoussi W.B. (2022). Germinal centre-driven maturation of B cell response to mRNA vaccination. Nature.

[42] Sureshchandra S., Lewis S.A., Doratt B.M., Jankeel A., Coimbra Ibraim I., Messaoudi I. (2021). Single-cell profiling of T and B cell repertoires following SARS-CoV-2 mRNA vaccine. JCI Insight.

[43] Goel R.R., Painter M.M., Apostolidis S.A., Mathew D., Meng W., Rosenfeld A.M., Lundgreen K.A., Reynaldi A., Khoury D.S., Pattekar A. (2021). mRNA vaccines induce durable immune memory to SARS-CoV-2 and variants of concern. Science.

[44] Guerrera G., Picozza M., D’orso S., Placido R., Pirronello M., Verdiani A., Termine A., Fabrizio C., Giannessi F., Sambucci M. (2021). BNT162b2 vaccination induces durable SARS-CoV-2–specific T cells with a stem cell memory phenotype. Sci. Immunol..

[45] Echaide M., Labiano I., Delgado M., Fernández de Lascoiti A., Ochoa P., Garnica M., Ramos P., Chocarro L., Fernández L., Arasanz H. (2022). Immune Profiling Uncovers Memory T-Cell Responses with a Th17 Signature in Cancer Patients with Previous SARS-CoV-2 Infection Followed by mRNA Vaccination. Cancers.

[46] von Massow G., Oh S., Lam A., Gustafsson K. (2021). Gamma Delta T Cells and Their Involvement in COVID-19 Virus Infections. Front. Immunol..

[47] Singh K., Cogan S., Elekes S., Murphy D.M., Cummins S., Curran R., Najda Z., Dunne M.R., Jameson G., Gargan S. (2022). SARS-CoV-2 spike and nucleocapsid proteins fail to activate human dendritic cells or γδ T cells. PLoS ONE.

[48] Schulien I., Kemming J., Oberhardt V., Wild K., Seidel L.M., Killmer S., Daul F., Lago M.S., Decker A., Luxenburger H. (2020). Characterization of pre-existing and induced SARS-CoV-2-specific CD8^+^ T cells. Nat. Med..

[49] Xiao C., Ren Z., Zhang B., Mao L., Zhu G., Gao L., Su J., Ye J., Long Z., Zhu Y. (2023). Insufficient epitope-specific T cell clones are responsible for impaired cellular immunity to inactivated SARS-CoV-2 vaccine in older adults. Nat. Aging.

[50] Khoury D.S., Cromer D., Reynaldi A., Schlub T.E., Wheatley A.K., Juno J.A., Subbarao K., Kent S.J., Triccas J.A., Davenport M.P. (2021). Neutralizing antibody levels are highly predictive of immune protection from symptomatic SARS-CoV-2 infection. Nat. Med..

[51] Soler M.F., Abaurrea A., Azcoaga P., Araujo A.M., Caffarel M.M. (2023). New perspectives in cancer immunotherapy: Targeting IL-6 cytokine family. J. Immunother. Cancer.

[52] Chen J., Wei Y., Yang W., Huang Q., Chen Y., Zeng K., Chen J. (2022). IL-6: The Link Between Inflammation, Immunity and Breast Cancer. Front. Oncol..

[53] Andreu-Ballester J.C., Cuéllar C., Colmena-Zaragoza J., Galindo-Regal L., Hurtado-Marcos C., González-Fernández J., Balciscueta Z., García-Ballesteros C., López-Chuliá F., Jiménez A.I. (2024). Anti-Anisakis antibodies in colon cancer patients and their relationship with γδ T-cells. Parasitol. Res..

[54] Andreu-Ballester J.C., Navarro A., Arribas M.A., Rico M., Albert L., García-Ballesteros C., Galindo-Regal L., Sorando-Serra R., López-Chuliá F., Peydro F. (2024). Increased Levels of Anti-Anisakis Antibodies During Hospital Admission in Septic Patients. Antibodies.

[55] Andreu-Ballester J.C., Hurtado-Marcos C., García-Ballesteros C., Pérez-Griera J., Izquierdo F., Ollero D., Jiménez A., Gil-Borrás R., Llombart-Cussac A., López-Chuliá F. (2025). Decreased gene expression of interleukin 2 receptor subunit γ (CD132) in tissues of patients with Crohn’s disease. World J. Gastroenterol..

[56] Field A.P. (2026). Discovering Statistics Using R and RStudio.

[57] Luke S.G. (2017). Evaluating significance in linear mixed-effects models in R. Behav. Res. Methods.

[58] Benjamini Y., Hochberg Y. (1995). Controlling the false discovery rate: A practical and powerful approach to multiple testing. J. R. Stat. Soc. Ser. B Stat. Methodol..

