# Supplementary material for: SARS-CoV-2 mRNA Vaccination Induces Reduced T-Cell Apoptosis in Patients with Solid Tumors
Source: Int J Mol Sci. 2026 Jul 10;27(14):6173. doi: 10.3390/ijms27146173 (PMC13411108; doi:10.3390/ijms27146173)
Supplement: Supplementary file 1 [file ijms-27-06173-s001.zip › ijms-4316788-supplementary.pdf]

Supplementary Materials.

Table S1. Clinical vaccination outcomes of the study population.

**Symptoms**	***N* = 39**
Injection-site pain	21 (53.85%)
Fatigue	15 (38.46%)
Body pain	11 (28.21%)
Fever	10 (25.64%)
Activity reduction	9 (23.08%)
Headache	6 (15.38%)
Injection-site erythema	5 (12.82%)
Loss of smell or/and taste	5 (12.82%)
Diarrhea	3 (7.69%)
Cough	2 (5.13%)
Sore throat	2 (5.13%)
Shortness of breath	1 (2.56%)
Vomiting	1 (2.56%)
**Outcomes**	
Medication intake (*)	13 (33.33%)
Medical consultation	2 (5.13%)
**Symptoms duration**	
24-48h	18 (46.15%)
3-7 days	6 (15.38%)
> 1 week	2 (5.13%)

Footnotes: *Antipyretic treatment

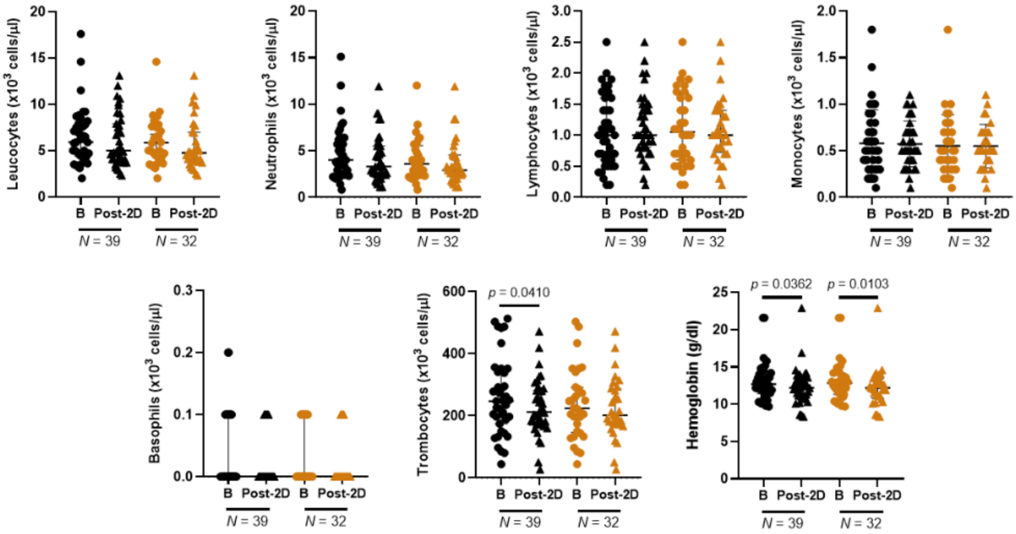

Figure S1. Additional hematological changes before and after SARS-CoV-2 vaccination. in the complete (N = 39) and restricted (N = 32) cohorts.

Results include leukocytes, neutrophils, lymphocytes, monocytes, basophils, thrombocytes and hemoglobin. Results are presented as individual values with median and interquartile range. Pairwise comparisons were performed using the Wilcoxon signed-rank test to determine the differences between Baseline (B) and Post-vaccination (Post-2D). Displayed p-values are unadjusted.

Table S2. Phenotypic definition of immune cell subsets.

Phenotype	Cell Population
Annexin V+7-AAD-	Early apoptotic cells
CD45+	Leukocytes
CD45+CD19+	B Lymphocytes
CD45+CD3+	T Lymphocytes
TCR PAN αβ+	αβ T Lymphocytes
TCR PAN γδ+	γδ T Lymphocytes
CD45+CD3+CD56+	Natural Killer T-like cells (NKT-like)
CD45+CD3-CD56+	Natural Killer Cells (NK)
CD45+CD3+CD4+	Helper T Lymphocytes
CD45+CD3+CD8+	Cytotoxic T Lymphocytes
CD45RA+CD62L+	Naïve
CD45RA+CD62L-	Terminal Differentiated Effector Memory RA+ (TEMRA)
CD45RA-CD62L+	Central Memory (CM)
CD45RA-CD62L-	Effector Memory (EM)

Footnotes: The selection of gating strategies for the phenotypic characterization of Natural Killer (NK) cells, B lymphocytes, αβ and γδ T-cell subsets was carried out following previously described methodologies [30,54-56]. Naïve, central memory (CM), effector memory (EM), and terminally differentiated effector memory RA^+ (TEMRA) T-cell subsets were defined according to CD45RA and CD62L expression patterns.

Table S3. Absolute counts of T-cell differentiation changes between Baseline (B) and Post-vaccination (Post-2D) time points in the complete (N = 39) and restricted (N = 32) cohorts.

Lymphocyte Subsets (x10³ cel/µl)			N = 39 B Median (IQR)	N = 39 Post-2D Median (IQR)	N = 39 Wilcoxon Z	N = 39 *p*-value	N = 39 *p*-value (FDR)	N = 32 B Median (IQR)	N = 32 Post-2D Median (IQR)	N = 32 Wilcoxon Z	N = 32 *p*-value	N = 32 *p*-value (FDR)
Total Lymphocytes			1.00 (0.60 - 1.50)	1.00 (0.80 - 1.50)	1.4846	0.1393	0.1885	1.05 (0.60 - 1.63)	1.00 (0.70 - 1.40)	0.3460	0.7371	0.8769
Total CD3+ T cells			0.57 (0.35 - 1.00)	0.76 (0.53 - 1.06)	1.8979	0.0586	0.1157	0.70 (0.35 - 1.07)	0.74 (0.43 - 1.03)	0.8041	0.4268	0.5774
Total CD3+CD4+			0.42 (0.19 - 0.60)	0.48 (0.26 - 0.64)	1.8979	0.0586	0.1157	0.43 (0.20 - 0.63)	0.40 (0.26 - 0.63)	0.8415	0.4054	0.5650
CD3+CD4+	Total	Naïve	0.09 (0.03 - 0.21)	0.15 (0.06 - 0.29)	2.3165	**0.0209**	0.0641	0.10 (0.04 - 0.21)	0.11 (0.05 - 0.25)	1.2715	0.2069	0.3525
		CM	0.15 (0.08 - 0.28)	0.22 (0.12 - 0.30)	1.9816	**0.0483**	0.1111	0.17 (0.08 - 0.30)	0.20 (0.11 - 0.27)	0.7293	0.4716	0.6174
		EM	0.07 (0.05 - 0.12)	0.07 (0.04 - 0.13)	0.5163	0.6105	0.6850	0.07 (0.05 - 0.12)	0.07 (0.04 - 0.13)	0.3366	0.7435	0.8769
		TEMRA	0.01 (0 - 0.01)	0.01 (0 - 0.01)	-1.0327	0.3050	0.3898	0.01 (0 - 0.01)	0.01 (0 - 0.01)	-1.1780	0.2425	0.3984
	αβ	Naïve	0.09 (0.03 - 0.21)	0.15 (0.06 - 0.35)	2.9445	**0.0033**	**0.0304**	0.10 (0.04 - 0.21)	0.11 (0.05 - 0.29)	2.0382	**0.0425**	0.2029
		CM	0.16 (0.08 - 0.28)	0.22 (0.12 - 0.30)	2.1491	**0.0322**	0.0779	0.17 (0.08 - 0.31)	0.20 (0.11 - 0.28)	1.0471	0.2994	0.4706
		EM	0.07 (0.05 - 0.12)	0.07 (0.04 - 0.12)	0.1116	0.9166	0.9483	0.07 (0.05 - 0.12)	0.07 (0.04 - 0.13)	-0.0187	0.9925	1.0000
		TEMRA	0.01 (0 - 0.01)	0.01 (0 - 0.01)	-0.7954	0.4304	0.5210	0.01 (0 - 0.01)	0.01 (0 - 0.01)	-0.7106	0.4832	0.6174
	γδ*		ND	ND	ND	ND	ND	ND	ND	ND	ND	ND
Total CD3+CD8+			0.22 (0.12 - 0.31)	0.26 (0.19 - 0.41)	2.6654	**0.0079**	0.0591	0.25 (0.13 - 0.32)	0.26 (0.15 - 0.40)	1.8138	0.0712	0.2089
CD3+CD8+	Total	Naïve	0.05 (0.01 - 0.09)	0.09 (0.04 - 0.14)	3.0003	**0.0028**	**0.0304**	0.06 (0.01 - 0.10)	0.06 (0.03 - 0.13)	1.9821	**0.0485**	0.2029
		CM	0.02 (0.01 - 0.03)	0.03 (0.02 - 0.05)	2.4421	**0.0149**	0.0591	0.02 (0.01 - 0.04)	0.03 (0.02 - 0.05)	1.7764	0.0772	0.2089
		EM	0.04 (0.02 - 0.05)	0.04 (0.02 - 0.07)	-0.0977	0.9277	0.9483	0.04 (0.02 - 0.05)	0.04 (0.02 - 0.07)	-0.4675	0.6469	0.8042
		TEMRA	0.06 (0.03 - 0.12)	0.08 (0.02 - 0.19)	1.7165	0.0874	0.1296	0.07 (0.03 - 0.13)	0.07 (0.02 - 0.19)	1.0097	0.3171	0.4706
	αβ	Naïve	0.05 (0.01 - 0.09)	0.08 (0.04 - 0.13)	3.0701	**0.0022**	**0.0304**	0.05 (0.01 - 0.09)	0.07 (0.04 - 0.13)	2.1504	**0.0323**	0.2029
		CM	0.02 (0.01 - 0.03)	0.03 (0.02 - 0.04)	2.4003	**0.0167**	0.0591	0.02 (0.01 - 0.04)	0.03 (0.01 - 0.04)	1.8138	0.0712	0.2089
		EM	0.04 (0.02 - 0.05)	0.04 (0.02 - 0.07)	0.2093	0.8396	0.9148	0.04 (0.02 - 0.06)	0.04 (0.02 - 0.08)	-0.0187	0.9925	1.0000
		TEMRA	0.06 (0.03 - 0.11)	0.07 (0.02 - 0.15)	1.5769	0.1164	0.1623	0.06 (0.03 - 0.12)	0.07 (0.02 - 0.18)	1.0097	0.3171	0.4706
	γδ	Naïve	0 (0 – 0)	0 (0 – 0)	3.6562	**0.0003**	**0.0121**	0 (0 - 0)	0 (0 - 0)	3.1040	**0.0020**	0.0906
		CM	0 (0 – 0)	0 (0 – 0)	1.8281	0.0686	0.1157	0 (0 - 0)	0 (0 - 0)	2.0008	**0.0464**	0.2029
		EM	0 (0 – 0)	0 (0 – 0)	1.6048	0.1101	0.1582	0 (0 - 0)	0 (0 - 0)	1.4398	0.1526	0.2996
		TEMRA	0 (0 - 0.01)	0 (0 - 0.01)	1.8700	0.0625	0.1157	0 (0 - 0.01)	0 (0 - 0.01)	1.9821	**0.0485**	0.2029
Total CD3+CD56+ (NKT-like)			0.05 (0.01 - 0.10)	0.06 (0.02 - 0.15)	2.4142	**0.0161**	0.0591	0.05 (0.02 - 0.09)	0.06 (0.02 - 0.15)	1.9821	**0.0485**	0.2029
CD3+CD56+ (NKT-like)	Total	Naïve	0 (0 - 0.01)	0.01 (0 - 0.02)	2.5817	**0.0100**	0.0591	0 (0 - 0.01)	0.01 (0 - 0.01)	1.8325	0.0683	0.2089
		CM	0	0	1.8421	0.0665	0.1157	0	0	1.4398	0.1526	0.2996

			(0 – 0)	(0 - 0.01)				(0 - 0)	(0 - 0.01)			
		EM	0.01 (0 - 0.02)	0.01 (0 - 0.03)	0.8931	0.3755	0.4669	0.01 (0 - 0.02)	0.01 (0 - 0.03)	0.2618	0.8007	0.9208
		TEMRA	0.03 (0.01 - 0.06)	0.03 (0.01 - 0.08)	2.2468	**0.0251**	0.0679	0.03 (0.01 - 0.06)	0.03 (0.01 - 0.08)	1.7951	0.0741	0.2089
	αβ	Naïve	0 (0 - 0.01)	0.01 (0 - 0.01)	2.4003	**0.0167**	0.0591	0 (0 - 0.01)	0.01 (0 - 0.01)	1.7016	0.0906	0.2193
		CM	0 (0 – 0)	0 (0 – 0)	1.8421	0.0665	0.1157	0 (0 - 0)	0 (0 - 0)	1.3837	0.1693	0.2996
		EM	0.01 (0 - 0.01)	0.01 (0 - 0.02)	1.2839	0.2016	0.2650	0.01 (0 - 0.01)	0.01 (0 - 0.02)	0.9536	0.3450	0.4960
		TEMRA	0.02 (0.01 - 0.04)	0.03 (0.01 - 0.06)	1.8002	0.0729	0.1157	0.02 (0.01 - 0.04)	0.02 (0.01 - 0.07)	1.4024	0.1636	0.2996
	γδ	Naïve	0 (0 – 0)	0 (0 – 0)	2.9724	**0.0030**	**0.0304**	0 (0 - 0)	0 (0 - 0)	2.2626	**0.0242**	0.2029
		CM	0 (0 – 0)	0 (0 – 0)	2.5306	**0.0116**	0.0591	0 (0 - 0)	0 (0 - 0)	2.4888	**0.0132**	0.2029
		EM	0 (0 – 0)	0 (0 – 0)	1.8002	0.0729	0.1157	0 (0 - 0)	0 (0 - 0)	1.4211	0.1580	0.2996
		TEMRA	0 (0 – 0.01)	0 (0 – 0.02)	2.3305	**0.0202**	0.0641	0 (0 - 0.01)	0 (0 - 0.01)	1.8886	0.0602	0.2089
Total CD3+CD4-CD8-			0.01 (0 - 0.02)	0.01 (0 - 0.04)	2.4142	**0.0161**	0.0591	0 (0 - 0.02)	0.01 (0 - 0.03)	2.4496	**0.0147**	0.2029
CD3+CD4-CD8-	γδ	Naïve	0 (0 – 0)	0 (0 – 0)	2.2607	**0.0242**	0.0679	0 (0 - 0)	0 (0 - 0)	1.4772	0.1421	0.2996
		CM	0 (0 – 0)	0 (0 – 0)	2.2049	**0.0280**	0.0714	0 (0 - 0)	0 (0 - 0)	2.0756	**0.0388**	0.2029
		EM	0 (0 – 0)	0 (0 - 0.01)	1.8002	0.0729	0.1157	0 (0 - 0)	0 (0 - 0.01)	1.7016	0.0906	0.2193
		TEMRA	0 (0 – 0.01)	0 (0 - 0.02)	1.7583	0.0799	0.1225	0 (0 - 0.01)	0 (0 - 0.01)	1.5146	0.1323	0.2996

Absolute counts of global, αβ and γδ T-cell differentiation subsets at baseline (B) and Post-vaccination (Post-2D). Differentiation stages were defined according to CD45RA and CD62L expression as naïve, central memory (CM), effector memory (EM), and terminal effector memory RA+ (TEMRA). Analyzed populations included CD3+CD4+ T cells, CD3+CD8+ T cells ,CD3+CD56+ NKT-like, and CD3+CD4−CD8− γδ T cells. Comparisons between baseline and post-vaccination values were performed using the Wilcoxon signed-rank test. To account for multiple comparisons, p-values were adjusted using the Benjamini–Hochberg false discovery rate (FDR) correction. *No CD3+CD4+ γδ T cells were detected. IQR: Interquartile range; ND: Not detected.

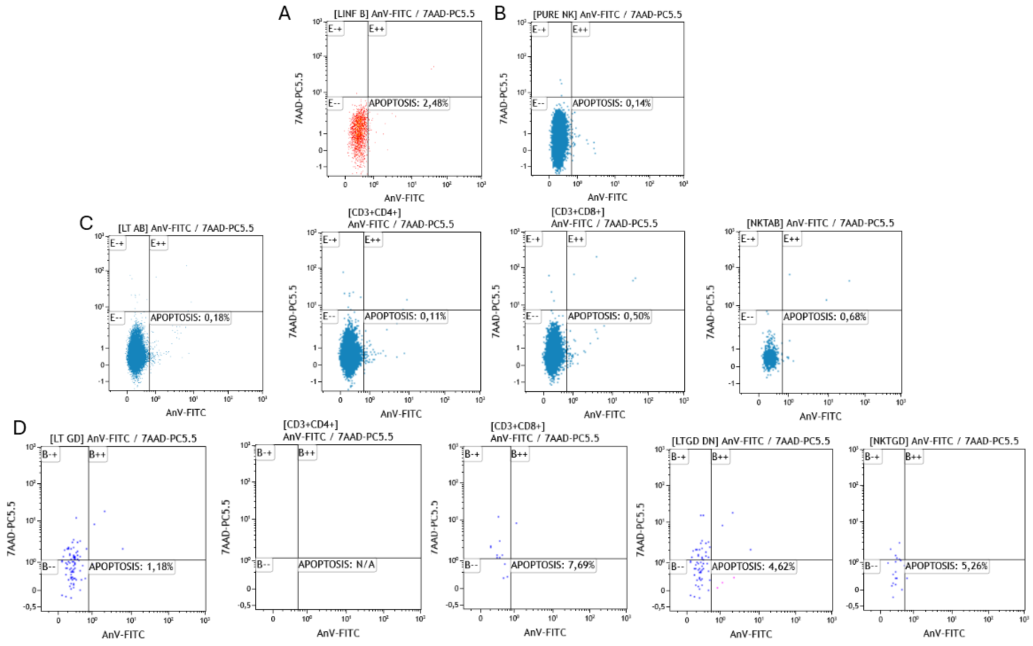

Figure S2. Representative Annexin V/7-AAD apoptosis analysis in lymphocyte subpopulations by flow cytometry.

Representative dot plots of gating strategy showing Annexin V-FITC and 7AAD-PC5.5 staining in different lymphocyte populations. Quadrant gates were established to distinguish viable cells (Annexin V−/7AAD−), **early apoptotic cells (Annexin V+/7AAD−)**, late apoptotic or secondary necrotic cells (Annexin V+/7AAD+), and dead cells (Annexin V−/7AAD+). (**A**) Apoptosis analysis in B lymphocytes (LINF B), (**B**) Classical NK cells (PURE NK). (**C**) Apoptosis analysis in αβ T-cell populations, including total αβ T lymphocytes (LT AB), CD3+CD4+ T cells, CD3+CD8+ T cells, and NKT-like αβ cells (NKTAB). (**D**) Apoptosis analysis in γδ T-cell populations, including total γδ T lymphocytes (LT GD), CD3+CD4+ γδ T cells, CD3+CD8+ γδ T cells, double-negative CD4-CD8- γδ T cells (LTGD DN), and NKT-like γδ cells (NKTGD). The γδCD4+ plot is included for completeness; however, this population was not detected in approximately 95% of the analyzed samples, resulting in negative or near-empty plots in most cases and precluding meaningful apoptosis quantification. Representative percentages for each population are indicated within the corresponding plots.

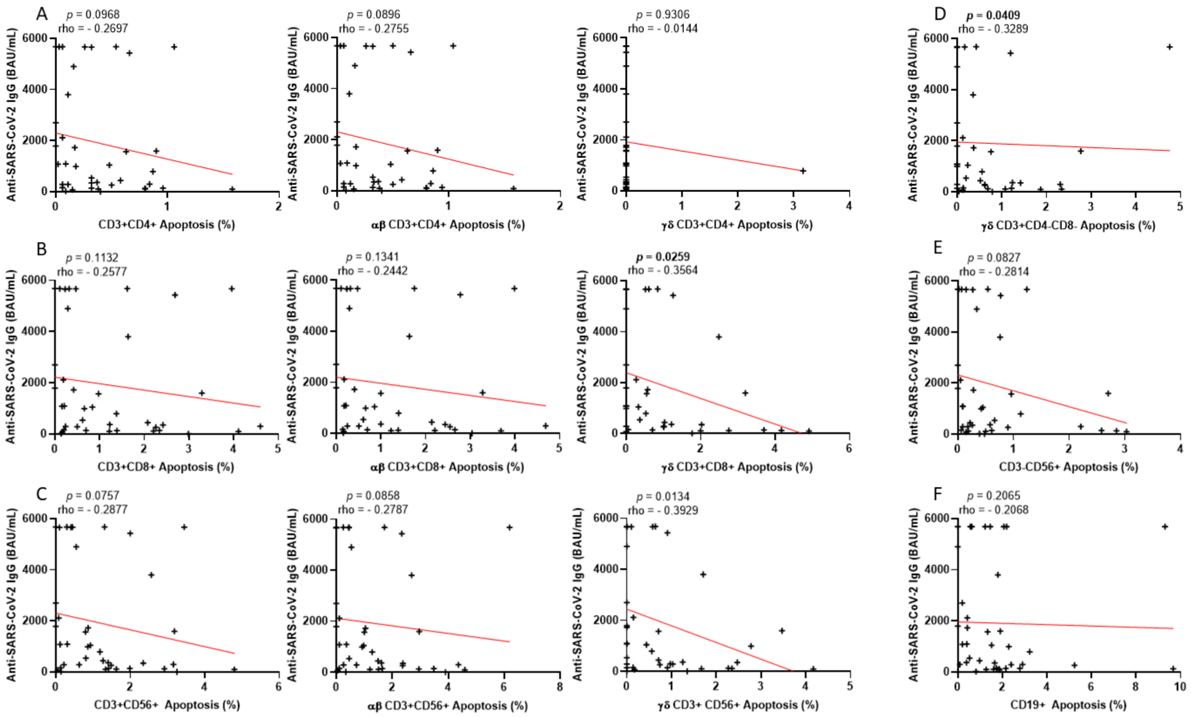

Figure S3A. Correlation between Post-vaccination (Post-2D) anti-Spike IgG production (BAU/mL) and apoptosis (%) of all the total, αβ and γδ immune cell populations (N = 39).
(**A**) CD4+ T cells. (**B**) CD8+ T cells. (**C**) CD3+CD56+ NKT-like cells. (**D**) CD3+CD4-CD8- γδ T cells. (**E**) CD3-CD56+ NK cells. (**F**) CD19+ B cells. Spearman's rho was used for correlation analysis. Displayed *p*-values are unadjusted.

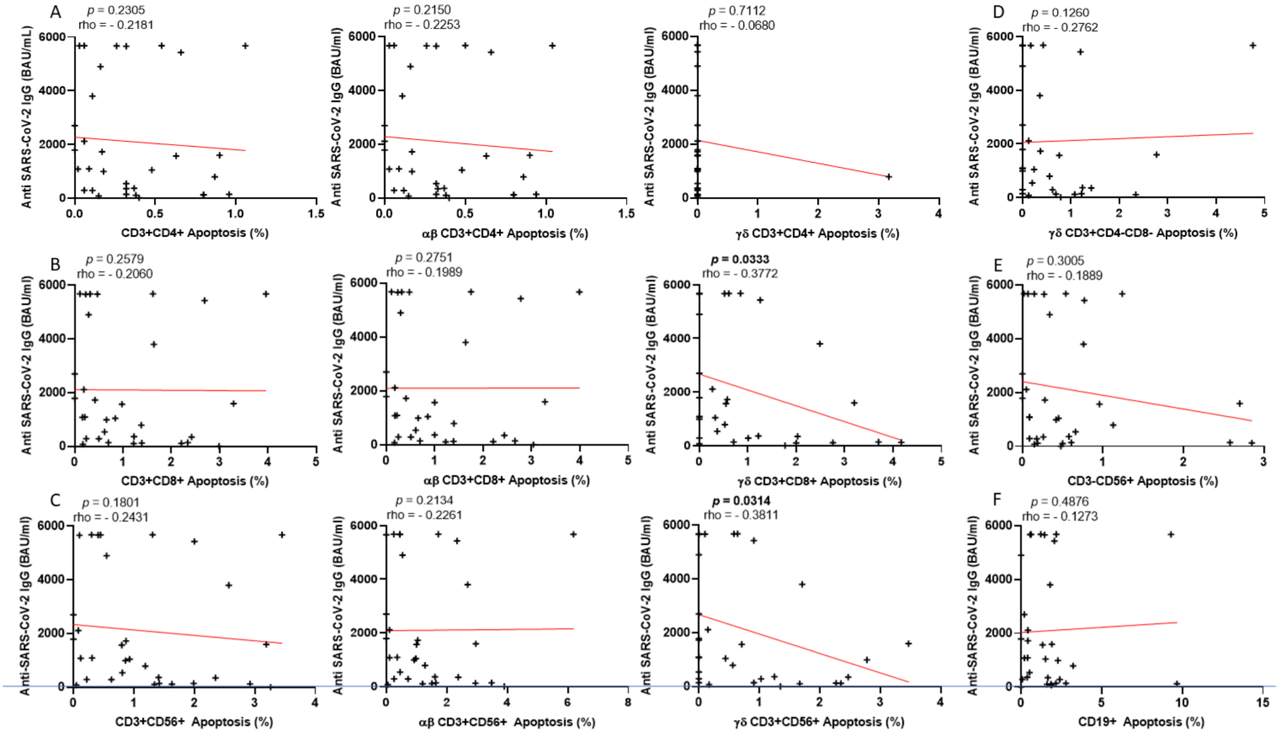

Figure S3B. Correlation between Post-vaccination (Post-2D) anti-Spike IgG production (BAU/mL) and apoptosis (%) of all the total, αβ and γδ immune cell populations in the restricted cohort (*N* = 32).

(**A**) CD4+ T cells. (**B**) CD8+ T cells. (**C**) CD3+CD56+ NKT-like cells. (**D**) CD3+CD4-CD8- γδ T cells. (**E**) CD3-CD56+ NK cells. (**F**) CD19+ B cells. Spearman's rho was used for correlation analysis. Displayed *p*-values are unadjusted.

Table S4. Flow cytometry antibody panel, clones, fluorochromes, catalog numbers and staining volumes used for immunophenotypic analysis.

Antibodies	Clone	Fluorochrome	Manufacturer	Catalog Nº	Volume/Test (µL)
Annexin V	-	FITC	Beckman Coulter	IM3614	10
7-AAD	-	7/AAD	Beckman Coulter	IM3614	20
TCR PAN αβ	IP26A	PE	Beckman Coulter	B49177	10
TCR PAN γδ	IMMU 510	PE	Beckman Coulter	B49176	10
CD19	J3-119	PE	Beckman Coulter	A07769	10
CD56	N901 (NKH-1)	PC7	Beckman Coulter	A21692	10
CD45	J33	Krome Orange (KrO)	Beckman Coulter	B36294	2.5
CD3	UCHT1	APC Alexa Fluor 700	Beckman Coulter	C86909	2.5
CD4	13B8.2	APC Alexa Fluor 750	Beckman Coulter	A94682	2.5
CD8	B9.11	Pacific Blue (PB)	Beckman Coulter	B49182	2.5
CD45RA	2H4LDH11 LDB9 (2H4)	ECD	Beckman Coulter	B49193	10
CD62L	DREG56	APC	Beckman Coulter	B30639	2.5

Footnotes: The selection of monoclonal antibodies and staining conditions was carried out following previously described methodologies [30,54-56].

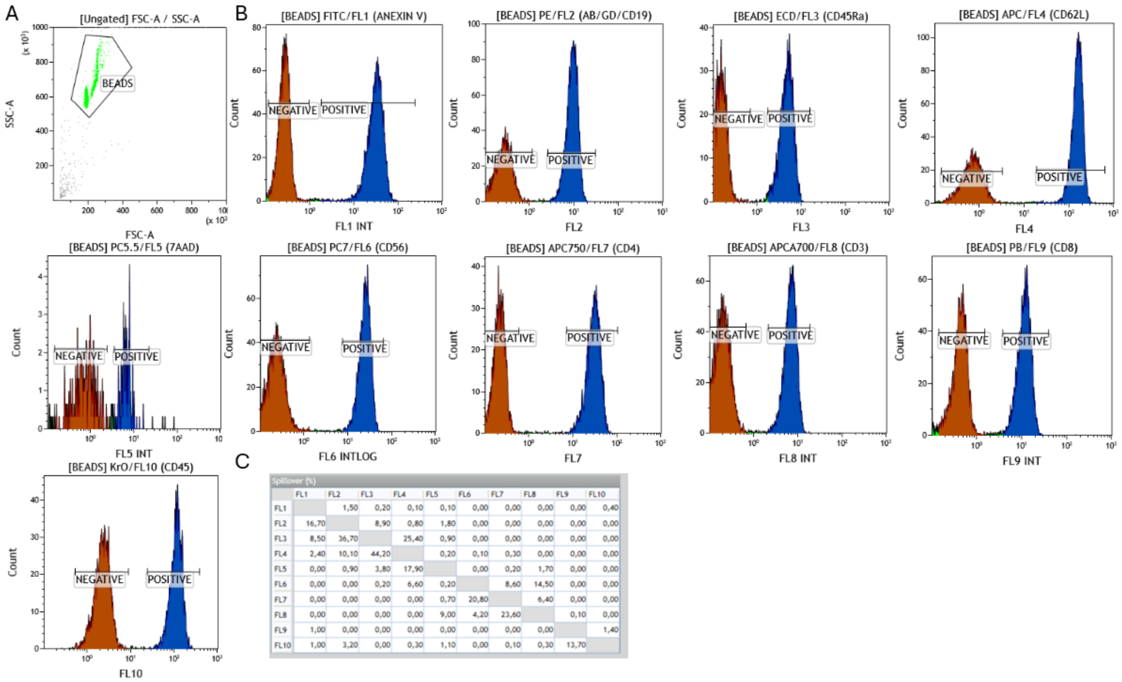

	FL1	FL2	FL3	FL4	FL5	FL6	FL7	FL8	FL9	FL10
FL1		1,50	0,20	0,10	0,10	0,00	0,00	0,00	0,00	0,40
FL2	16,70		8,90	0,80	1,80	0,00	0,00	0,00	0,00	0,00
FL3	8,50	36,70		25,40	0,90	0,00	0,00	0,00	0,00	0,00
FL4	2,40	10,10	44,20		0,20	0,10	0,30	0,00	0,00	0,00
FL5	0,00	0,90	3,80	17,90		0,00	0,20	1,70	0,00	0,00
FL6	0,00	0,00	0,20	6,60	0,20		8,60	14,50	0,00	0,00
FL7	0,00	0,00	0,00	0,00	0,70	20,80		6,40	0,00	0,00
FL8	0,00	0,00	0,00	0,00	9,00	4,20	23,60		0,10	0,00
FL9	1,00	0,00	0,00	0,00	0,00	0,00	0,00	0,00		1,40
FL10	1,00	3,20	0,00	0,30	1,10	0,00	0,10	0,30	13,70	

Figure S4. Representative compensation controls and compensation matrix used for multicolor flow cytometry analysis.

(**A**) VersaComp beads (Beckman Coulter, Inc) were stained individually with each fluorochrome-conjugated antibody included in the panel to generate single-color compensation controls. The upper-left panel shows the FSC-A versus SSC-A gate used to identify VersaComp beads prior to compensation analysis. (**B**) Representative histograms show negative and positive bead populations acquired for FITC/FL1 (Annexin V), PE/FL2 (TCRαβ or TCRγδ and CD19), ECD/FL3 (CD45RA), APC/FL4 (CD62L), PC5.5/FL5 (7-AAD), PC7/FL6 (CD56), APC-A750/FL7 (CD4), APC-A700/FL8 (CD3), Pacific Blue(PB)/FL9 (CD8), and Krome Orange/FL10 (CD45). Two compensation control tubes were prepared using the same fluorochrome combinations and detector settings: one for the TCRαβ panel and one for the TCRγδ panel. The only difference between both tubes was the FL2 channel, which contained a PE-conjugated anti-TCRαβ antibody in the αβ panel and a PE-conjugated anti-TCRγδ antibody in the γδ panel, respectively. Histograms were displayed on logarithmic scale and used to calculate fluorescence spillover between channels. (**C**) The corresponding spillover/compensation matrix generated from these controls is shown in the lower panel and was applied to all subsequent analyses of Annexin V/7-AAD apoptosis assays and immunophenotyping experiments.

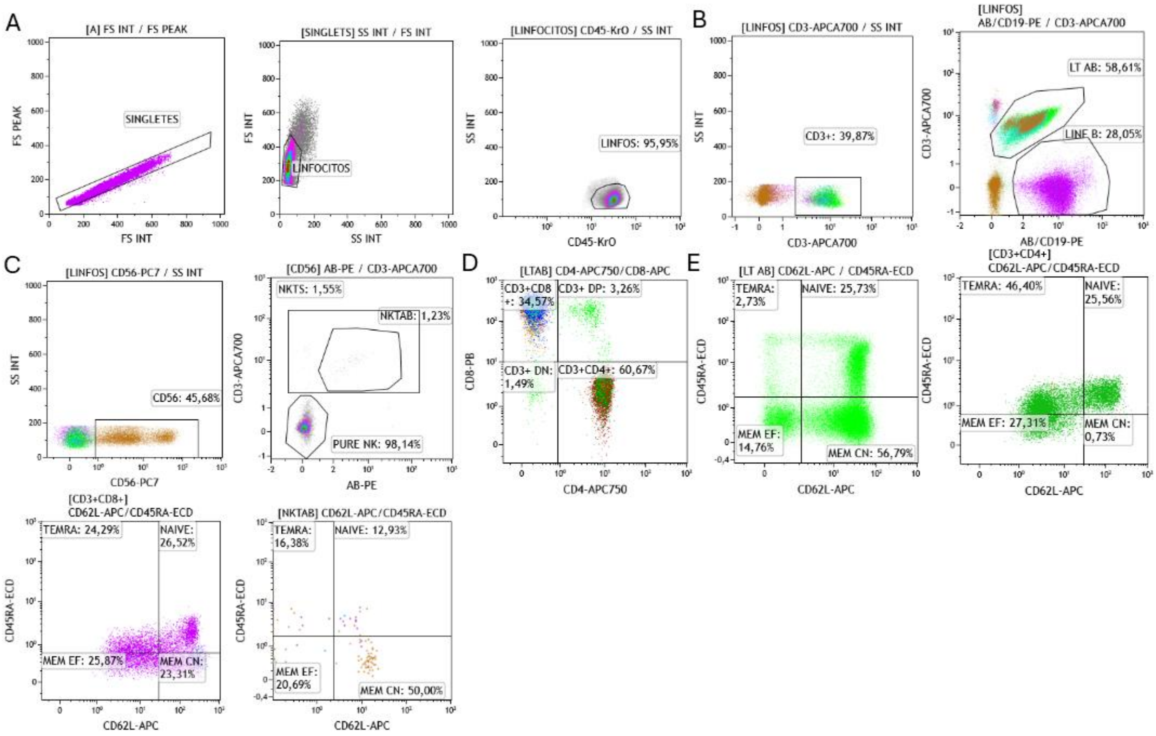

Figure S5. Representative gating strategy for identification of $\alpha\beta$ T-cell subpopulations, NK cells, and B lymphocytes by multicolor flow cytometry.

(**A**) Sequential gating was performed starting with exclusion of doublets using FS INT versus FS PEAK parameters, followed by singlet selection and lymphocyte identification based on CD45-KrO expression. (**B**) CD3+ T lymphocytes were subsequently identified and separated into TCR$\alpha\beta$-positive lymphocytes (LT AB) and B lymphocytes (LINF B) according to AB-PE and CD3-APC-A700 expression. (**C**) CD56 expression was used to identify NK and NKT-like populations. (**D**) Within the $\alpha\beta$ T-cell compartment, CD4+ and CD8+ T-cell subsets were defined using CD4-APC750 and CD8-Pacific Blue staining. (**E**) Differentiation subsets were characterized according to CD62L-APC and CD45RA-ECD expression as naïve (CD62L+CD45RA+), central memory (MEM CN; CD62L+CD45RA−), effector memory (MEM EF; CD62L−CD45RA−), and terminally differentiated effector memory RA-positive cells (TEMRA; CD62L−CD45RA+). Representative percentages for each gated population are indicated within the corresponding plots.

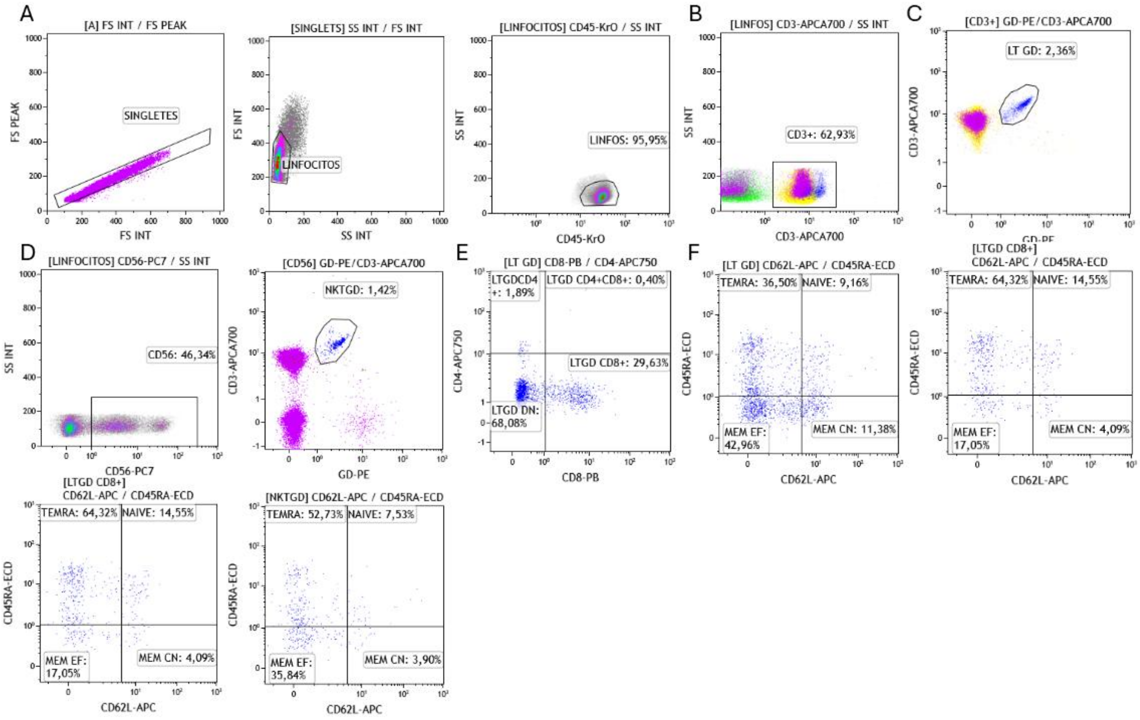

Figure S6. Representative gating strategy for identification of γδ T-cell subpopulations by multicolor flow cytometry.

(**A**) Sequential gating strategy used to identify lymphocyte populations, including singlet selection (FS INT vs. FS PEAK), lymphocyte gating based on forward and side scatter properties, and $CD45^+$ leukocyte identification. (**B**) Selection of $CD3^+$ T lymphocytes from total lymphocytes. (**C**) Identification of $TCR\gamma\delta^+$ T cells within the $CD3^+$ population. (**D**) Analysis of CD56 expression and identification of the $CD3^+TCR\gamma\delta^+CD56^+$ (NKT-like γδ T-cell) subset. (**E**) Distribution of γδ T cells according to CD4 and CD8 expression. The majority of γδ T cells displayed a $CD4^-$ phenotype, predominantly comprising $CD4^-CD8^-$ and $CD4^-CD8^+$ subsets. Notably, $CD4^+$ γδ T cells were not detected in approximately 95% of the analyzed samples, indicating that this population was exceedingly rare in the study cohort. (**F**) Differentiation status of γδ T-cell subsets based on CD45RA and CD62L expression, defining naïve ($CD45RA^+CD62L^+$), central memory ($CD45RA^-CD62L^+$), effector memory ($CD45RA^-CD62L^-$), and terminally differentiated effector memory (TEMRA; $CD45RA^+CD62L^-$) populations.

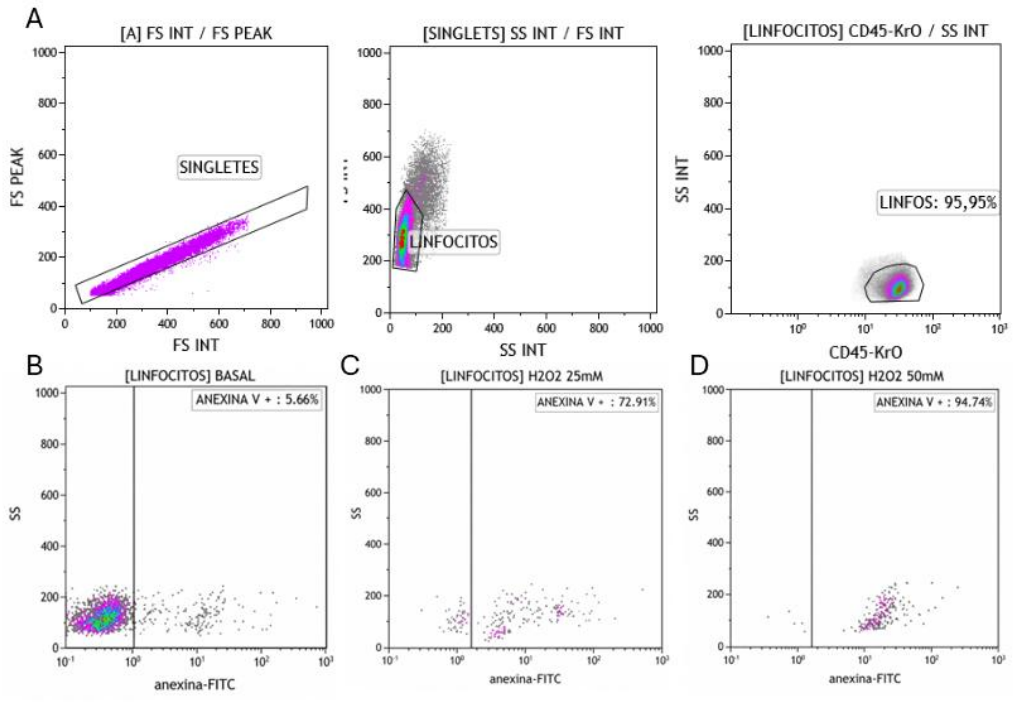

Figure S7. Representative gating strategy and Annexin V-FITC control used to establish the positivity threshold for apoptosis analysis.

(**A**) Sequential gating was performed by excluding doublets using FS INT versus FS PEAK parameters, followed by singlet selection and lymphocyte identification according to CD45-KrO expression and side scatter characteristics. (**B**) Representative Annexin V-FITC staining plots obtained under basal conditions and after hydrogen peroxide (H_2O_2) stimulation: (**C**) 25 mM and (**D**) 50 mM were used to define the cutoff for Annexin V positivity. Increasing Annexin V positivity after oxidative stress induction confirmed the appropriateness of the selected cutoff for subsequent apoptosis analyses.

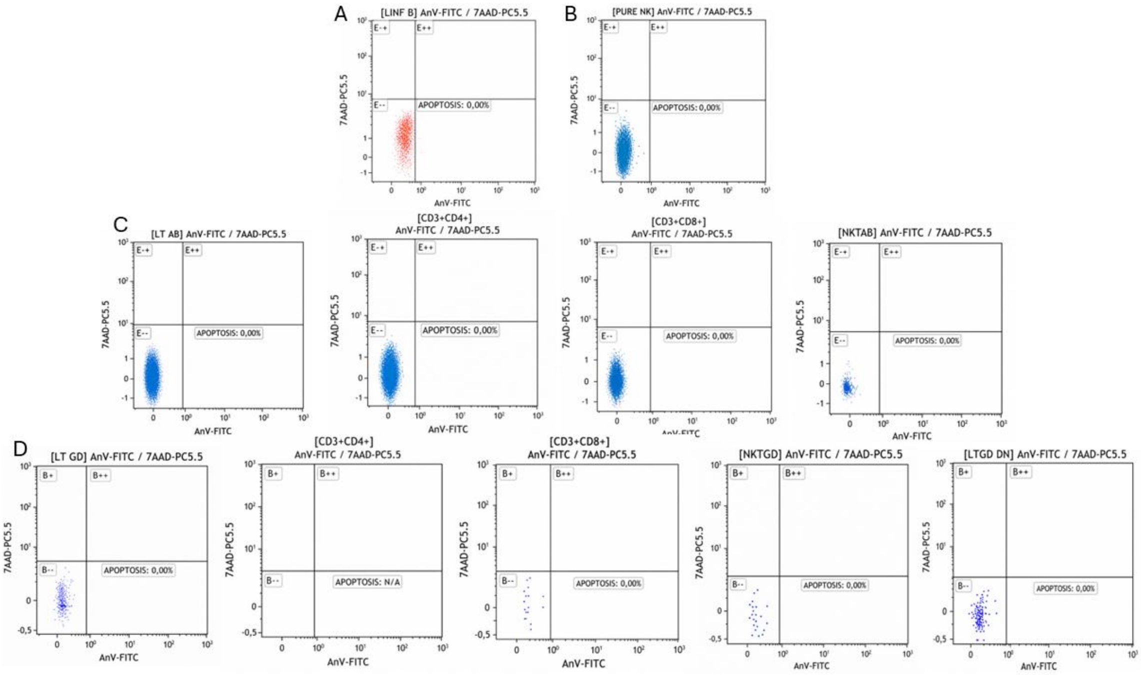

Figure S8. Representative unstained Annexin V/7-AAD controls lymphocyte subpopulations. Representative unstained control dot plots showing background fluorescence levels for Annexin V-FITC and 7AAD-PC5.5 staining in different lymphocyte populations. Unstained controls for (**A**) B lymphocytes (LINF B). (**B**) Unstained controls for classical Natural Killer cells (PURE NK). (**C**) Unstained controls for αβ T-cell populations, including total αβ T lymphocytes (LT AB), CD3+CD4+ T cells, CD3+CD8+ T cells, and NKT-like αβ cells (NKTAB). (**D**) Unstained controls for γδ T-cell populations, including total γδ T lymphocytes (LT GD), CD3+CD4+ γδ T cells, CD3+CD8+ γδ T cells, double-negative CD4-CD8- γδ T cells (LTGD DN), and NKT-like γδ cells (NKTGD).
